# Analysis of Plant–Plant Interactions Reveals the Presence of Potent Antileukemic Compounds

**DOI:** 10.3390/molecules27092928

**Published:** 2022-05-04

**Authors:** David E. Mery, Amanda J. Compadre, Paola E. Ordóñez, Edward J. Selvik, Vladimir Morocho, Jorge Contreras, Omar Malagón, Darin E. Jones, Philip J. Breen, Michael J. Balick, Flavio G. Gaudio, Monica L. Guzman, Cesar M. Compadre

**Affiliations:** 1 Department of Pharmaceutical Sciences, University of Arkansas for Medical Sciences, Little Rock, AR 72205, USA; dmery@uams.edu (D.E.M.); amanda.compadre@ucsf.edu (A.J.C.); eselvik@uams.edu (E.J.S.); dejones@uams.edu (D.E.J.); breenphilipj@uams.edu (P.J.B.); 2 SeqRX, LLC., Little Rock, AR 72205, USA; 3 School of Chemical Sciences and Engineering, Yachay Tech University, Urcuquí 100119, Ecuador; pordonez@yachaytech.edu.ec; 4 Departamento de Química, Universidad Técnica Particular de Loja, San Cayetano Alto s/n, Loja 110107, Ecuador; svmorocho@utpl.edu.ec (V.M.); omalagon@utpl.edu.ec (O.M.); 5 Department of Medicine, Division of Hematology/Oncology, Weill Cornell Medical College, New York, NY 10021, USA; joc4006@med.cornell; 6 Institute for Economic Botany, New York Botanical Garden, New York, NY 10458, USA; mbalick@nybg.org; 7 Department of Emergency Medicine, New York Presbyterian-Weill Cornell Medicine, New York, NY 10065, USA; flg9002@med.cornell.edu

**Keywords:** antileukemic activity, allelopathic activity, sesquiterpene lactones, plant–plant interactions, anticancer compound screening, traditional medicine

## Abstract

A method to identify anticancer compounds in plants was proposed based on the hypothesis that these compounds are primarily present in plants to provide them with an ecological advantage over neighboring plants and other competitors. According to this view, identifying plants that contain compounds that inhibit or interfere with the development of other plant species may facilitate the discovery of novel anticancer agents. The method was developed and tested using *Magnolia grandiflora*, *Gynoxys verrucosa*, *Picradeniopsis oppositifolia*, and *Hedyosmum racemosum*, which are plant species known to possess compounds with cytotoxic activities. Plant extracts were screened for growth inhibitory activity, and then a thin-layer chromatography bioautography assay was conducted. This located the major antileukemic compounds **1**, **2**, **4**, and **5** in the extracts. Once the active compounds were located, they were extracted and purified, and their structures were determined. The growth inhibitory activity of the purified compounds showed a significant correlation with their antileukemic activity. The proposed approach is rapid, inexpensive, and can easily be implemented in areas of the world with high biodiversity but with less access to advanced facilities and biological assays.

## 1. Introduction

Natural products are an invaluable source of novel chemical structures for drug development. Approximately 32% of the small molecules approved for cancer treatment are natural or naturally derived from organisms such as plants, bacteria, and marine species [1]. Natural products have been evolutionarily optimized with many sp^3^-hybridized carbons, chiral centers, and complex ring structures [2,3]. It is very unlikely that complex naturally occurring compounds, such as homoharringtonine, paclitaxel, and vincristine, could have emerged from a drug design program [4].

At present, there is no comprehensive explanation as to why plants contain anticancer compounds, nor is there a paradigm to offer clues on how to locate plants likely to contain anticancer compounds. This presents a challenge when deciding which plants to investigate from the more than 390,000 vascular plant species reported to inhabit Earth [5]. Two of the most commonly used approaches to select plants to study are the traditional medicine approach and the biodiversity approach.

Traditional systems of medicine, such as Ayurveda, Unani, Kampo, and traditional Chinese medicine, have thrived for thousands of years and have provided valuable insights, leading to the discovery of new treatments for many ailments [6,7,8]. Traditional medicine has been more helpful in identifying treatments for conditions such as malaria that present symptoms such as fever, nausea, and vomiting; that are identifiable before treatment; and that disappear following successful treatment [9]. Unfortunately, the link between anticancer therapies and the traditional use of their source plants is not as clear as in other diseases. In what has been described as monumental work, Jonathan Hartwell’s book, titled *Plants Used Against Cancer* (1982), including extended work (2000), contains documentation, personal testimony, and traditional uses for over 3000 plant species that have been reported for treating cancer [10,11]. However, with the exception of etoposide and teniposide [12], there are no clinically used drugs available for cancer treatment that have directly emerged from, or have been significantly linked to, this compilation. One proposed explanation is that the term “cancer” is poorly defined in these accounts, or is only loosely associated with visible or tangible conditions such as warts, polyps, tumors, etc. [12]. The diagnosis and treatment of cancer requires sophisticated techniques that go beyond visible and tangible inspection, such as magnetic resonance imaging, computed tomography, positron emission tomography, or blood sample analysis [13,14,15]. These techniques are not available to traditional healers, making the traditional medicine approach a largely unreliable method for selecting anticancer plants.

The biodiversity approach has been the most successful collection method for finding anticancer compounds with clinical relevance, such as paclitaxel, camptothecin, podophyllotoxin, and the vinca alkaloids [16,17,18,19]. This process begins with the randomized collection of plants, followed by the use of standard extraction procedures [20]. Multiple cycles of activity-guided fractionation are performed using cancer bioassays until a compound with cytotoxic activity is identified. This randomized approach is notorious for producing a low yield of promising agents when compared with approaches based on the screening of synthetic compound libraries. Therefore, it is often performed on a large scale, which makes the process costly, time-consuming, and environmentally harmful, since large amounts of waste solvent are generated [4,21].

As an addition to the current approaches, we propose that the discovery of anticancer compounds could be expedited by using an ecological approach. This approach is based on the hypothesis that anticancer compounds are primarily present in plants to provide them with an ecological advantage by interfering with the signaling or metabolic pathways of competing species. This approach has various strengths that facilitate the search for anticancer compounds in a much more rational, systematic way.

Plants are stationary and cannot escape their environments. This leaves them susceptible to herbivores, bacteria, viruses, fungi, and competitor plants present in the immediate vicinity. Plants must also retain the ability to survive variable environmental pressures such as radiation, water loss, heat, salinity, and nutrient deprivation [22,23]. Despite lacking sophisticated immune systems, plants have been quite successful at surviving these conditions. It has been noted that plants accumulate many secondary metabolites [24]. Secondary metabolites are not essential for growth, development, or reproduction, but are released by plants under different conditions and by different processes. They enter the soil through root exudation, decomposition of leaf and root tissue, and as trichome leachates [25]. Many secondary metabolites alter the germination, growth, and reproduction of neighboring plants by interacting with and altering critical cellular processes.

The ability of a plant species to chemically inhibit a neighboring plant through the release of such secondary metabolites is called allelopathy [26,27]. The accumulation and release of these allelopathic secondary metabolites, called allelochemicals, may influence their survival, the dominance and succession of plants, community formation, the biodiversity of a region, and crop production [24,26,28]. Allelochemicals, which have a major role in chemical defense, must have a level of inherent bioavailability to successfully leave the donor plant, travel through the soil, and enter the receiving plant [29].

Although allelopathy is a process common to plants, it has been argued that allelopathy is more effective when and where plants are experiencing stress [30,31]. Notably, the concentrations of various clinically useful anticancer compounds in plants increase when the plant experiences some form of moderate stress. This has been reported for camptothecin [32], paclitaxel [33], vinblastine [34], podophyllotoxin [35], and several sesquiterpene lactones with known cytotoxic properties [36,37,38,39,40,41].

Identifying plants containing compounds that inhibit or interfere with the development of other plant species may increase the likelihood of discovering compounds with anticancer activity in a time- and cost-effective manner. To test this hypothesis, four species were selected: *Magnolia grandiflora* L, *Gynoxys verrucosa* Wedd., *Picradeniopsis oppositifolia* (Nutt.) Rydb. ex Britton, and *Hedyosmum racemosum* (Ruiz & Pav.) G. Don. These plant species have been reported to contain compounds with activity against cancer cell lines [42,43,44,45]. They are from three unrelated plant families (Magnoliaceae, Asteraceae, and Chloranthaceae), they grow in different plant habitats, and were available in sufficient amounts from previous collections.

**Figure 1:**
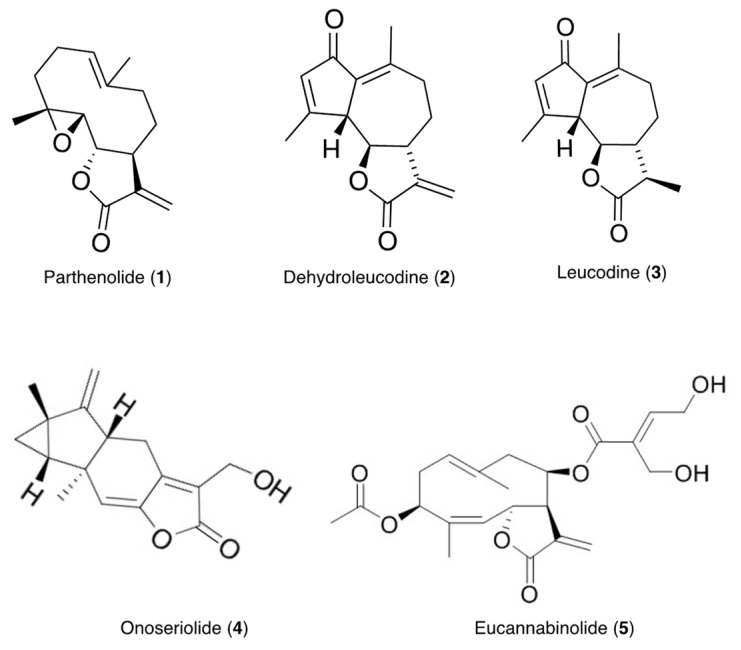
Structures of compounds isolated from the tested plants.

The presence of compounds that inhibit or interfere with the development of other plant species on the extracts of the test plants was investigated using seeds of *Lolium perenne* L. ssp. *multiflorum* (Lam.) Husnota, a representative monocot, and *Lactuca sativa* L., a representative dicot. The use of plant seeds has several advantages over standard cytotoxicity assays with cell lines. Seeds are multicellular eukaryotic test systems that experience spatial and temporal changes in their cellular development. The seeds of these plants germinate uniformly and rapidly, and are commonly used for allelopathy studies [46,47,48]. The inhibitory effects of the extracts and compounds **1**–**5** (Figure 1) isolated from the extracts were contrasted and correlated with their antileukemic activity using MV4-11 (acute myeloid leukemia, AML) cells.

## 2. Results and Discussion

To determine whether the extracts inhibited or interfered with the development of other plant species, dose–response assays were performed for the plant extracts against *Lolium perenne* L. ssp. *multiflorum* and *Lactuca sativa* L., and the concentration that produced 50% growth inhibition (GI_50_) was calculated. The antileukemic activity of extracts and compounds was measured using MV4-11 (AML) cells, and the concentration that produced a 50% reduction in cell viability (LD_50_) was calculated.

Thin-layer chromatography (TLC) bioautography has been used extensively in the literature for isolating antibiotic or antifungal compounds from plant extracts [49,50,51,52]. The TLC bioautography assay works well with bacteria and fungi, since very little material is needed for activity and the resolution is high, with the organism covering the entire plate like a lawn. Plant seeds are larger than bacteria and fungi, which limits the number of seeds that can be used. Additionally, seedlings may shift the positions of their roots into other zones when growing. To address these issues, we developed a stainless-steel plant grid with symmetrical rows that was inserted into the agar after contact bioautography. The plant grid limited the diffusion of extracts in the agar and confined seeds to rows. This allowed for easy identification of the active fractions on the TLC plate since the position of the active row of the plant grid could be accurately translated into a measured zone on the TLC plate.

*Magnolia grandiflora* L. is a common evergreen tree that is native to the southeastern United States [53]. *M. grandiflora* contains compounds that have plant inhibitory activity and anticancer activity and was used as a positive control when developing the method.

Plants have difficulty growing under the canopy of *Magnolia grandiflora*, and the chipped mulch of these trees has been shown to reduce the germination and growth of *Lactuca. sativa* [54]. *M. grandiflora* has been reported to contain inhibitory sesquiterpene lactones in its leaves that contribute to its ability to suppress the growth of competing plants [55,56]. Marin and Mansilla [57] reported that *M. grandiflora* extracts had antileukemic activity. Parthenolide, a constituent of *M. grandiflora*, has been reported to have strong antileukemic effects on both leukemia stem and progenitor cells [37,58].

**Figure 2:**
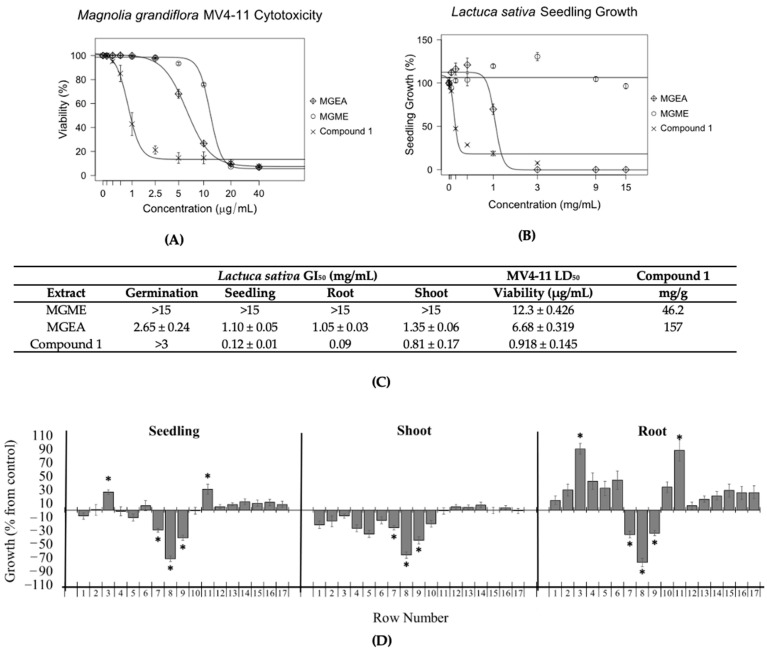
The inhibitory effects on seedlings by *Magnolia grandiflora* extracts (**A**) and their MV4-11 cytotoxicity (**B**) correlated with the concentration of compound **1**. (**C**) GI_50_ and LD_50_ values correlated with the concentration of compound **1** in the extracts. The bioautography assay (**D**) confirmed that compound **1** was the major inhibitory component. (**A**) Inhibitory effects of M. grandiflora ethyl acetate (MGEA) and methanolic (MGME) extracts on the seedlings of *L. sativa*. Experiments were performed in triplicate. (**B**) Concentration–response curves of MGEA and MGME extracts against MV4-11 cell lines after 48 h of incubation. Experiments were performed in duplicate, and parthenolide (compound **1**) was used as a positive control. (**C**) Extract and compound **1** GI_50_ values for *L. sativa* and LD_50_ values for MV4-11, as well as the ratio (mg/g) of compound **1** in extracts. (**D**) Bioautography assay of MGEA extract on the growth of *L. sativa*. Growth is expressed as the % difference from the control value, and the chromatographic bands are divided by 1 cm rows from the application point to the elution limit on the TLC plate (1–17). * The level of significance was *p* ≤ 0.05. Row 8 was identified as compound **1**. All data are presented as the mean ± SEM.

The aerial parts of *Magnolia grandiflora* were extracted with 70% methanol/water (MGME) or ethyl acetate (MGEA). These extracts were tested against *Lolium perenne* and *Lactuca sativa* to measure their effects on the germination and growth inhibition of seedlings, roots, and shoots (Figure 2A,C and Figure S2). MGEA extract displayed GI_50_ values for the germination and growth of *L*. *perenne* and *L. sativa* seedlings, roots, and shoots at concentrations under 3 mg/mL (Figure 2A and Figure S2). The MGME extract was only effective at reducing the growth of *L. perenne* roots (Figure S2). The antileukemic activity of the extracts was tested against MV4-11 leukemia cells at concentrations of 0 to 40 µg/mL. MGEA was found to be more potent against MV4-11 leukemia cells than the MGME extract (Figure 2B,C).

TLC bioautography assays were carried out with *Lactuca sativa* seeds (Figure 2D). The reason for this was that the seeds of *L. sativa* are smaller, have less size variability, and grow faster than the seeds of *Lolium perenne*. The overall effect on the seedling was used to determine the positions of the active compounds in the TLC plate. Data in different rows had unequal variances when the *F* test was employed. Thus, the Welch correction was applied to the *t*-test to analyze the data. The results are presented as percentage differences from the control. Zero represents the control, positive values represent stimulation, and negative values represent inhibition.

*Magnolia grandiflora* extracts were tested with 13.5 mg MGEA and 30 mg MGME for each assay (Figure 2C and Figure S2). These amounts were determined by running small TLC plates with different concentrations of extract to identify the maximum amount that produced distinct bands under UV inspection at both 254 and 365 nm. Compound **1**, which was identified as parthenolide, was the major inhibitory component in both the MGEA extract (Figure 2D) and the MGME extract (Figure S3).

The amount of compound **1** in the extracts was determined by gas chromatography/mass spectrometry (GCMS) analysis using the sesquiterpene lactone dehydroleucodine (DHL) as an internal standard. The MGEA and MGME extracts contained 157 and 46 mg of compound **1** per gram of dry extract, respectively, indicating an increase of approximately 3.4-fold in the amount of compound **1** in the ethyl acetate extract (Figure 2C). These results showed that the activity level of the extracts on the plant seeds and on the MV4-11 cells paralleled the concentration of compound **1** in the corresponding extracts (Figure 2A–C).

To test the validity and scope of the method, it was applied to *Gynoxys verrucosa* V.M. Badillo and *Hedyosmum racemosum* (Ruiz & Pav.) G. Don. These plant species were collected in the province of Loja in Ecuador, and we did not observe, nor have there been reports, of them having plant inhibitory activities. However, they have been reported to contain antileukemic compounds [43,44]. The method was also applied to *Picradeniopsis oppositifolia* (Nutt) Rydb. Ex Britt. This plant species was collected in the state of Montana in the United States. There are no reports of this species having plant inhibitory effects. However, we observed that in the hot and arid regions where the plant was collected, it grows in populations that are clearly separated from neighboring vegetation. *P. oppositifolia* has been reported to contain antileukemic compounds [45].

**Figure 3:**
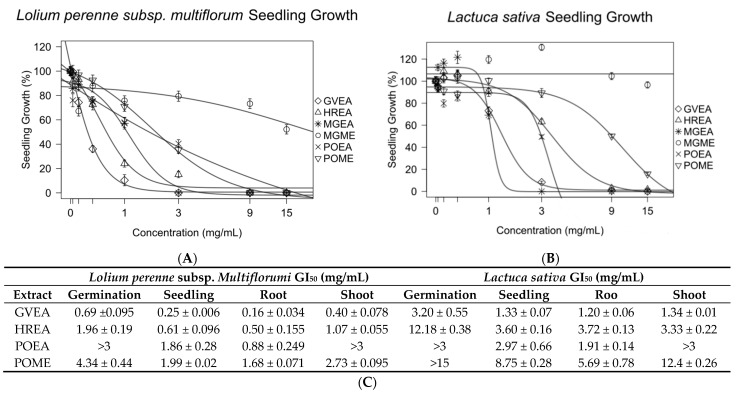
Extracts from plants known to have antileukemic compounds showed strong inhibitory effects on the seedlings of *Lolium perenne* (**A**) and *Lactuca sativa* (**B**). Experiments were conducted in triplicate. The concentrations that produced 50% growth inhibition (GI_50_) of germination and the growth of seedlings, roots, and shoots were calculated from a nonlinear regression of concentration–response models. All data are presented as the mean ± SEM (**C**).

*Gynoxis verrucosa* was extracted with ethyl acetate (GVEA), *Hedyosmum racemosum* was extracted with ethyl acetate (HREA), and *Picradeniopsis oppositifolia* was extracted with 70% methanol/water (POME) or ethyl acetate (POEA). These extracts were tested against *Lolium perenne* and *Latuca sativa* to identify germination or growth inhibition of seedlings, roots, and shoots (Figure 3 and Figure S3). The GI_50_ of all extracts against seedlings of *L. perenne* was under 2 mg/mL. Apart from POME, the GI_50_ of the extracts against the seedling *L. sativa* was less than 4 mg/mL. Overall, *L. perenne* was found to be more sensitive than *L. sativa.* POEA was found to be more potent against both test seeds than POME.

**Figure 4:**
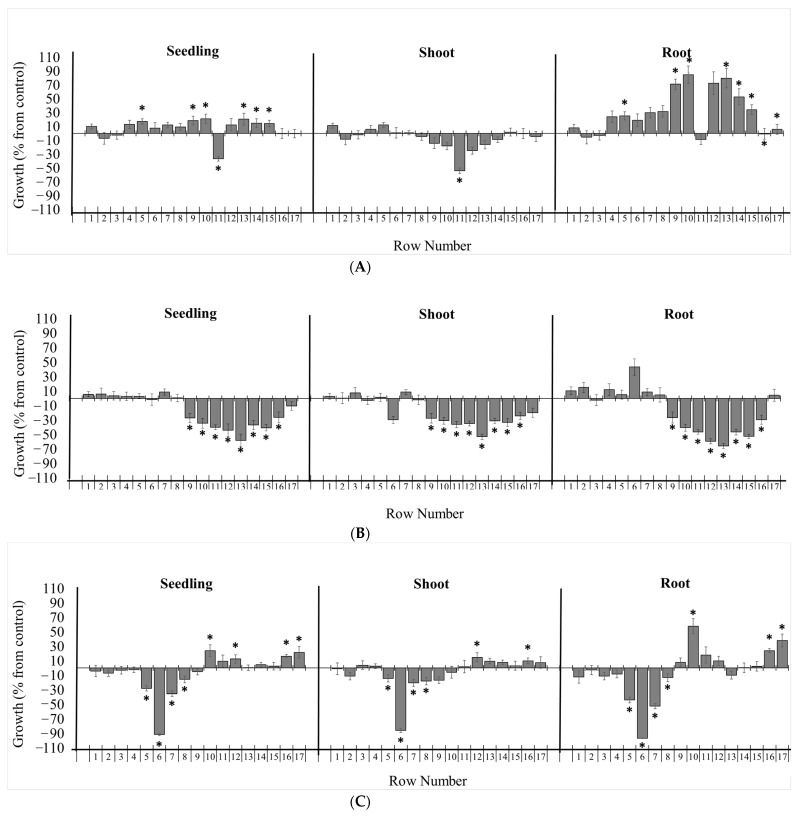
Effects of chromatographic fractions of on the growth of *Lactuca sativa*. Bioautography assays of (**A**) GVEA, (**B**) HRMEA, and (**C**) POEA extracts on the growth of *L. sativa*. The bioautography assay of Gynoxys verrucosa extract revealed dehydroleucodine as the major inhibitory component (row 11). The bioautography assay of *Hedyosmum racemosum* extract revealed onoseriolide as the major inhibitory component (row 13). The bioautography assay of *Picradeniopsis oppositifolia* extract revealed eucannabinolide as the major inhibitory component (row 6). Growth is expressed as the % difference from the control, and the chromatographic bands were divided by 1 cm rows from the application point to the elution limit on the TLC plate (1–17). * Significance is indicated at *p* ≤ 0.05. All data are presented as the mean ± SEM.

**Figure 5:**
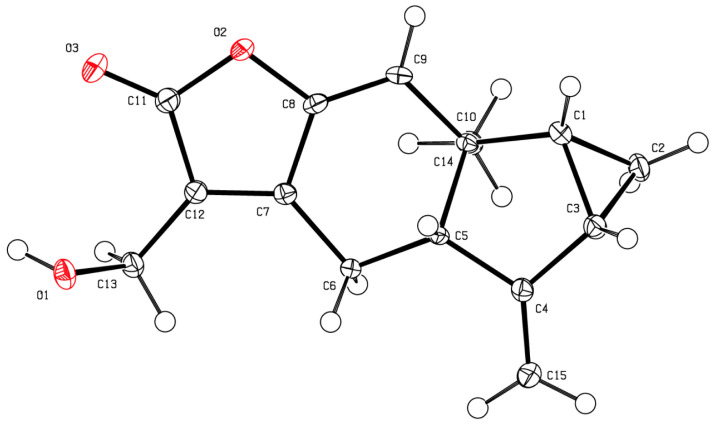
A 3D-ORTEP projection of the X-ray crystal structure of Compound 4 with 50% probability ellipsoids.

Bioautography assays of *Picradeniopsis oppositifolia* with 70% methanol/H_2_O (POME) and ethyl acetate (POEA) extract, *Gynoxys* *verrucosa* ethyl acetate (GVEA) aerial extract, and *Hedyosmum racemosum* aerial extract were carried out with *Lactuca sativa* seeds (Figure 4). The *Gynoxys* *verrucosa* extract (6.5 mg of GVEA) was submitted to the bioautography assay. Row 11 significantly inhibited seedling length (Figure 4A). This inhibition was due to a reduction in the length of the shoot but not the root. Row 11 was separated and analyzed and shown to contain two compounds. These compounds were purified and recrystallized from ethyl acetate, and their structures were established to be dehydroleucodine (**2**) and leucodine (**3**) [43]. The *Hedyosmum* *racemosum* extract (HREA, 13.5 mg) was submitted to bioautography assays. Rows 9 to 16 significantly inhibited seedling, root, and shoot lengths (Figure 4B). The inhibition was centered around row 13, with a broad band potentially indicating diffusion. Row 13 was separated, analyzed, and found to consist of a single compound. This compound was purified and recrystallized from a mixture of methanol:chloroform by the slow evaporation method, and its structure was established to be onoseriolide (**4**) by X-ray crystallography (Figure 5).

The extracts of *Picradeniopsis oppositifolia* (POEA 15 mg; POME 36 mg) were submitted for the bioautography assay (Figure 4C and Figure S4). The POEA extract inhibited seedling growth on rows 5 to 8 (Figure 4C). Both the roots and shoots of the seedling showed significant growth inhibition in rows 5 to 8. The POME extract was shown to stimulate seedling growth in rows 5, 6, and 10 (Figure S4). For both extracts, row 6 was separated and analyzed, and was found to consist of one compound. This compound was purified as an amorphous solid that was identified by NMR and LC/MS as eucannabinolide (**5**).

**Figure 6:**
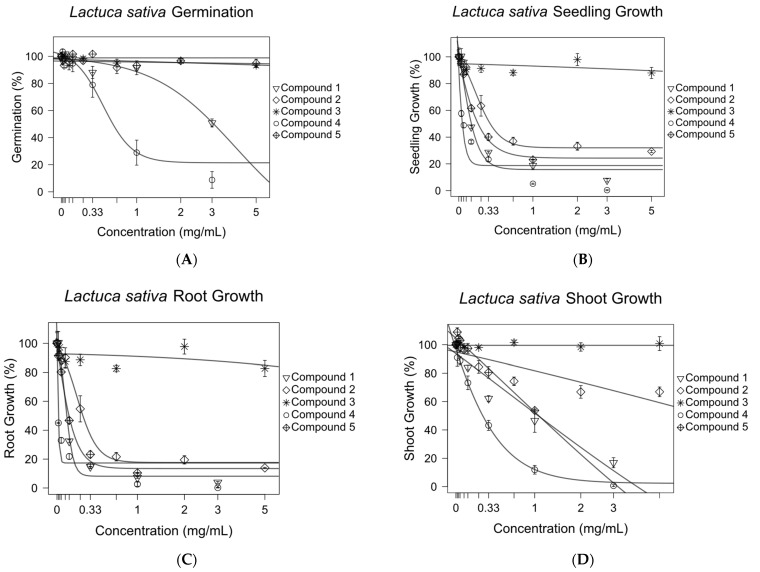
Inhibitory effects of purified antileukemic compounds on the germination (**A**) and growth of the *Lactuca sativa* seedlings (**B**), roots (**C**), and shoots (**D**). Sigmoidal concentration–response models were used to calculate the growth inhibition at 50% (GI_50_) for compounds 1–5 against *L. sativa* root, shoot, and seedling growth. The *optim* function in R was used to fit nonlinear regression curves. Error bars are ±SEM.

**Figure 7:**
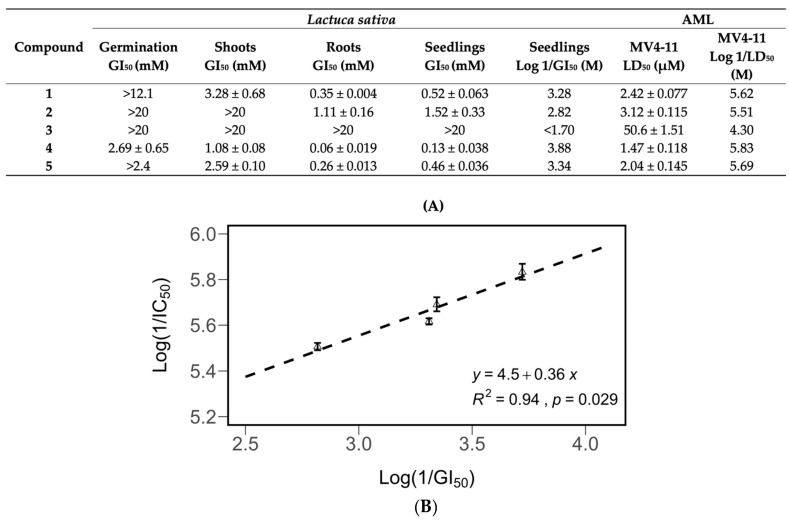
Inhibitory potency of compounds 1–5 against the growth and germination of *Lactuca sativa* seedlings, and their cytotoxic potency (Log 1/LD_50_) against MV4-11 leukemia cells (**B**). Correlation between the cytotoxic potency and the inhibitory potency (**A**). All data are presented as the mean ± SEM. Compound 3 was not included in the derivation of the equation.

The effects of compounds **1**–**5** on the germination and seedling, root, and shoot growth of *Lactuca sativa* were tested in a Petri dish assay. Concentration–response curves were generated for all compounds (Figure 6). All compounds except compound **3** were shown to inhibit 50% of the growth of *L. sativa* seedlings at concentrations of 1.52 mM or lower (Figure 7). The roots were more sensitive to pure compounds, with 50% of the growth being inhibited at concentrations of 1.11 mM or lower (Figure 7). The shoots were less sensitive. They were not inhibited by compound **2** at concentrations of 20 mM. Compound **1**, being the third most active growth inhibitor, inhibited 50% of the growth of seedlings and roots at 0.52 and 0.35 mM, but was much less effective against shoots, inhibiting 50% of their growth at 3.28 mM.

The growth inhibitory activity of the purified compounds showed a significant correlation with their antileukemic activity. The compounds were tested for antileukemic activity against MV4-11 leukemia cells. The GI_50_ values for *Lactuca sativa* were converted into Log (1/GI_50_) values, and the LD_50_ values for MV4-11 were converted into Log (1/LD_50_) values. These values were compared in a scatterplot, and correlations between seedling inhibition, antileukemic activity (Figure 7), and root leukemic activity (Figure S5) were discovered.

**Figure 8:**
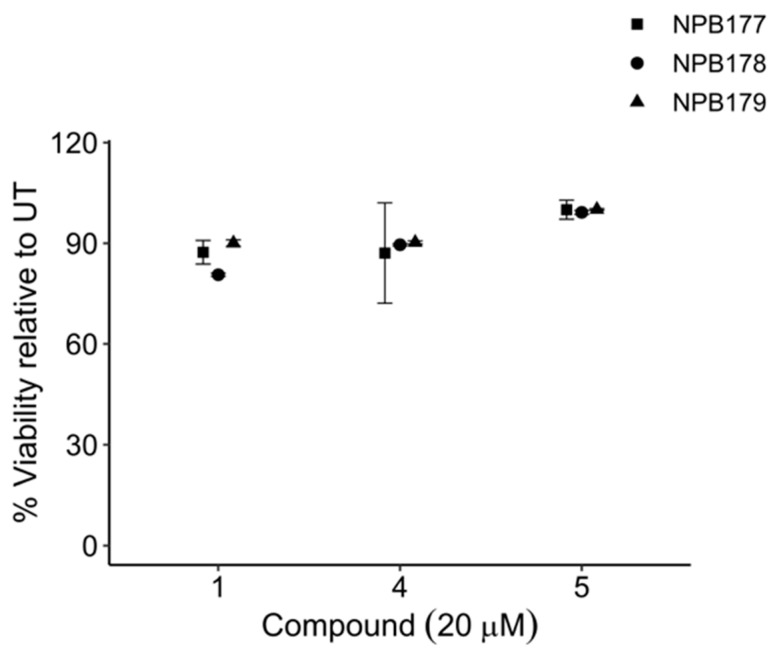
Viability of normal cells after treatment with compounds 1, 4, and 5 compared with untreated control cells (UT). Peripheral blood mononuclear cells (PBMCs) were isolated from the buffy coats of healthy adult volunteers (NPB 177, NPB 178, NPB 179) and treated for 48 h with 20 μM of compounds 1, 4, or 5. Cells that were negative for YO-PRO-1 iodide and 7-AAD were scored as viable. Error bars are ±SEM.

Compounds **1**, **4**, and **5** were tested against normal peripheral blood mononuclear cells (PBMCs) isolated from healthy adult volunteers (Figure 8). At the highest concentration tested, 20 μM, which was 6 times higher than their LD_50_s against leukemia cells (Figure 7), the percentage of relative viability (treated cells/untreated cells ± SEM) for compounds 1, 4, 5 was 86.0 ± 1.53%, 89.0 ± 2.54%, and 99.8 ± 0.50%, respectively. Compound **3** has been reported to show significantly less toxicity against normal cells than against leukemia cell lines [43]. These results supported the notion that the proposed approach was capable of discovering compounds that target tumor cells without causing significant harm to noncancerous cells.

## 3. Materials and Methods

### 3.1. Plant Materials

The leaves and stems of *Magnolia grandiflora* L. were collected in October 2017 from Little Rock, AR, USA (Compadre 131 barcode: 02921345). *Picradeniopsis oppositifolia* (Nutt) Rydb. Ex Britt. was collected in Albion, MT (J. McCarthy 100, barcode 2921342). Voucher specimens were identified and deposited at the William and Lynda Steere Herbarium, New York Botanical Garden. *Gynoxys verrucosa* Wedd. was collected from Yangana (Voucher PPN-as-11), and *Hedyosmum racemosum* (Ruiz & Pav.) G. Don was collected from El Tiro (Voucher HUTPL14285). Both locations are in the Loja Province of Ecuador, and collections were conducted with permission from the Ministry of Environment, Water and Ecological Transition of Ecuador under registry number MAE-DNB-CM-2016-0048. Herbarium specimens were deposited at the Herbarium of the Applied Chemistry Institute of the Universidad Tecnica Particular de Loja, Ecuador.

### 3.2. Extraction and Preparation of Extracts

Samples of the aerial parts of the plants studied were air-dried and ground into a powder and then macerated in an orbital shaker (25 °C and 250 rpm). The extracts were concentrated at a reduced pressure while keeping the temperature below 30 °C. *Magnolia grandiflora* samples (100 g) were extracted with 800 mL of either ethyl acetate (MGEA) or 70% methanol/water (MGME) to yield 5.98 g and 23.78 g of MGEA and MGME extracts, respectively. These extracts were used for the MV4-11 assays and to measure the concentration of compound **1**. Another sample of *M grandiflora* (3.65 kg) from the same collection was extracted with ethyl acetate in the same manner as above to yield 372.4 g of crude extract. A portion of this extract was filtered through a reverse-phase C18 bed (LiChroprep Merck 25–40 μm) with 70% methanol/water to remove the chlorophyll, a process that helped to improve the tumor cells without causing significant harm to noncancerous cells. resolution of the TLC bioautography assays. MGME was redissolved in methanol and filtered to remove methanol insoluble compounds, which also improved the resolution of the TLC bioautography assay.

A sample of *Picradeniopsis oppositifolia* was extracted with ethyl acetate (a 260 g sample) or 70% methanol/water (a 50 g sample). The ethyl acetate extract was dissolved in methanol/water and filtered through a reverse-phase C18 bed, as described above, to yield 14.5 g of dried extract (POEA). The methanolic/water extract was suspended in methanol and filtered to yield 10.0 g of extract (POME).

A sample of *Gynoxys verrucosa* was extracted with ethyl acetate, dissolved, and filtered through a reverse-phase C18 bed, as indicated above, to yield 6.48 g of crude extract (GVEA).

A sample of *Hedyosmum recemosum* (500 g) was macerated (20–25 °C) for 2 h successively with hexane and ethyl acetate. The ethyl acetate extract was filtered and concentrated under reduced pressure at 35 °C to yield 9.47 g of dried extract (HREA).

### 3.3. Bioassay for Germination and Growth Studies

Seeds of ryegrass (*Lolium perenne* L. ssp. *multiflorum* (Lam.) Husnota) and lettuce (*Lactuca sativa* L.) were treated with 2% NaOCl for 15 min and 2 min, respectively. Seeds smaller than 5.0 mm for *L. perenne* or 3.0 mm for *L. sativa* and/or seeds that were discolored were discarded. All experiments were conducted in triplicate, and the seeds were incubated at 22–25 °C with a 12 h photoperiod (LED, 4000 K). The lettuce seeds were incubated in the dark for 48 h before starting the photoperiod. Lettuce seeds were incubated for 6 days, and ryegrass was incubated for 7 days. After incubation, plates were stored at 5 °C to avoid subsequent growth during the measurement process. Germination was determined for each treatment, and the root and shoot lengths were recorded using a computer-interfaced digital caliper system package (500-171-30, Gaging and Software Technologies, Inc., Colorado Springs, CO, USA) comprising a CD-6” AX Mitutoyo caliper, a 600-520-KB-USB computer interface, and a C-FS25-06 data send switch.

Extracts were dissolved in methanol, applied to filter paper (Whatman No. 1) circles (35 mm diameter) that were dried, and placed in 6-well plates. Then, 0.6 mL of 2-(N-morpholino)-ethanesulfonic acid (MES) buffer (0.01 M pH 6.0) was added to each well. The extracts were tested at 0, 0.03, 0.11, 0.33, 1, 3, 9, and 15 mg/mL against both test species using 15 seeds in each well.

Pure compounds were dissolved in methanol and applied to filter paper circles (90 mm diameter) that were dried and placed in Petri dishes. Then, 1.5 mL of deionized water was added to each plate. Compounds **1**, **4**, and **5** were tested at 0, 0.01, 0.03, 0.11, 0.33, and 1 mg/mL, and compounds **2** and **3** were tested at 0, 0.02, 0.07, 0.22, 0.67, and 2 mg/mL against lettuce seeds using 20 seeds in each dish.

Data were analyzed statistically using Welch’s test with the significance level set at 0.05. The results are presented as the percentage difference from the control, which was given a value of zero. Positive values represent stimulation, and negative values represent inhibition. The *optim* function in R [59] was used to fit logistic functions to root and shoot inhibition (% inhibited) from log-transformed (log10(x)) concentration values for each pure compound. GI_50_ (growth inhibition at 50% of control growth) were calculated, and ±SEM was used for error bars.

### 3.4. Contact Bioautography Assay

Sterile agar sheets were prepared by pouring 100 mL of a 0.75% *w*/*v* agar solution buffered with MES (0.01 M pH 6.0) into 25 × 25 cm square petri dishes (Nunc 240835 Square Culture Dish, Thermo Scientific, Waltham, MA, USA). Plates were solidified at 4 °C and equilibrated at 25 °C for 30 min before contact bioautography.

Precoated glass (0.250 mm thick, 20 × 20 cm area) thin-layer chromatography (TLC) plates (Analtech Silica Gel GF F254, Newark, DE, USA) were eluted with ethyl acetate-methanol (2:1) to remove possible interference from contaminants in the plates. Dried plant extracts were dissolved in methanol (0.05–0.3 mg/mL) and applied to the plates with a glass capillary tube (50 µL). After sample application, the plates were dried at 40 °C for 1 h and then placed in a vacuum chamber for 30 min. MGEA was eluted with ethyl acetate/hexane (6:4). POEA was eluted with acetone/hexane (6:4). GVEA and HREA were eluted twice with ethyl acetate/hexane (7:3). After elution, the plates were dried for 1 h at 40 °C and then placed in a vacuum chamber for 30 min. The dried TLC plates were placed facedown onto the agar sheets to imprint for 45 min at 5 °C, after which the agar sheets were separated from the TLC plates and the plant grid was secured over the agar sheets, aligning the bottom of the grid with the application line on the TLC plate. Subsequently, 20 *Lactuca sativa* seeds were placed in each of the 17 rows of the plant grid. The agar sheets were incubated at 22–25 °C in a microbiological incubator. Seeds were grown using the same photoperiod. After 5 days, plants were stored at 5 °C to avoid growth during the measurement process. Germinated seeds were counted for each treatment, and the root and shoot lengths were recorded as described above.

**Figure 9:**
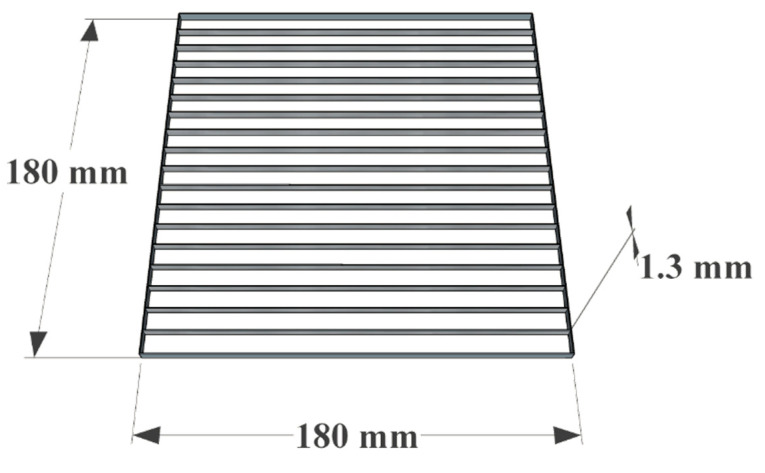
Plant grid used for the TLC bioautography assays.

The plant grid was custom built (Wescon Machining Inc.1325 Thomas G Wilson Dr, Conway, AR 72032, USA) from a solid 3/16-inch stainless-steel plate with a water jet cutter. The dimensions of the grid were 180 × 180 mm × 47.6 mm (L × W × H), and the thickness of the divider walls was 1.3 mm. The grid covered the TLC bioautography plate above the application line, dividing the plate into 18 rows with the grid walls tall enough to keep the plants from crossing over into other zones (Figure 9).

### 3.5. Isolation and Characterization of Compounds 1–5

Duplicate TLC plates with MGEA, POEA, GVEA, and HREA extracts were eluted under the same conditions as the TLC plates used for the bioautography assays. Areas corresponding to the major inhibition zones identified in the bioautography assays (MGEA row 8, POEA row 6, GVEA row 11, and HREA row 13) were marked on the duplicate plates. Silica was scraped from the marked areas, transferred to a flask, and stirred with ethyl acetate (3×). Then, the ethyl acetate extracts were combined, filtered, and concentrated at reduced pressure. The isolated compounds (1–5) were tentatively identified by their mass spectra and 1D-NMR. Additional amounts of compounds **1**–**5** were isolated using silica gel column chromatography with hexane–ethyl acetate gradients, and with the samples separated by TLC used as references. The identities of compounds **1**–**3** and **5** were confirmed by MS and 1D- and 2D-NMR to be parthenolide [60], dehydroleucodine [43], leucodine [43], and eucannabinolide [45], respectively. MS, 1D- and 2D-NMR, and X-ray crystallography were used to determine the identity of compound **4** as onoseriolide [44].

**Parthenolide (1)** white crystals, mp = 116 °C, **^1^H NMR (400 MHz, CDCl_3_)** δ 6.31 (d, *J* = 3.7 Hz, 1H), 5.60 (d, *J* = 3.3 Hz, 1H), 5.19 (ddd, *J* = 12.4, 3.8, 1.5 Hz, 1H), 3.83 (t, *J* = 8.6 Hz, 1H), 2.81 − 2.71 (m, 2H), 2.47 − 2.30 (m, 2H), 2.23 − 2.07 (m, 4H), 1.77 − 1.63 (m, 4H), 1.28 (d, *J* = 0.7 Hz, 3H), 1.27 − 1.17 (m, 1H); **^13^C NMR (101 MHz, CDCl_3_)** δ 169.38, 139.39, 134.73, 125.42, 121.36, 82.59, 66.53, 61.65, 47.81, 41.35, 36.50, 30.79, 24.28, 17.41, 17.09; **MS (ESI+):** C_15_H_20_O_3_ [M + H]^+^: 249.19.

**Dehydroleucodine (2)** white crystals, mp = 129 °C,**^1^H NMR (400 MHz, CDCl_3_)** δ 6.20 − 6.13 (m, 2H), 5.46 (d, *J* = 3.1 Hz, 1H), 3.61 (t, *J* = 10.0 Hz, 1H), 3.54 − 3.46 (m, 1H), 2.94 − 2.82 (m, 1H), 2.56 − 2.46 (m, 1H), 2.46 − 2.42 (m, 3H), 2.42 − 2.33 (m, 1H), 2.31 (t, *J* = 1.1 Hz, 3H), 2.26 − 2.15 (m, 1H), 1.50 − 1.36 (m, 1H); **^13^C NMR (101 MHz, CDCl_3_)** 195.94, 169.63, 169.29, 152.01, 138.64, 135.80, 132.06, 119.00, 84.48, 53.15, 53.02, 37.34, 24.51, 21.91, 19.94, **MS (ESI+):** C_15_H_16_O_3_ [M + H]^+^: 245.17.

**Leucodine (3)** white crystals mp = 199 °C, **^1^H NMR (400 MHz, CDCl_3_)** δ 6.42 (*p*, *J* = 1.3 Hz, 1H), 3.88 (t, *J* = 10.0 Hz, 1H), 3.71 − 3.63 (m, 1H), 2.74 − 2.63 (m, 4H), 2.63 − 2.57 (m, 1H), 2.57 − 2.54 (m, 3H), 2.54 − 2.44 (m, 1H), 2.31 − 2.15 (m, 2H), 1.69 − 1.54 (m, 1H), 1.53 (d, *J* = 6.9 Hz, 3H), **^13^C NMR (101 MHz, CDCl_3_)** δ 196.07, 177.70, 170.07, 152.26, 135.71, 132.02, 84.36, 56.53, 52.72, 41.29, 37.71, 26.14, 21.77, 19.97, 12.44; **MS (ESI+):** C_15_H_18_O_3_ [M + H]^+^: 247.21.

**Onoseriolide** (**4**): This compound was dissolved in a mixture of methanol and chloroform and left out to crystallize by slow evaporation of the solvent to obtain white crystals. X-ray data of compound **4**: crystal data: C_15_H_16_O_3_, Mw = 244.28, space group P21, a = 7.2254(2) Å, b = 5.7254(1)Å, c = 14.3199(4)Å, V = 593.76 (3) Å3, Z = 2, T = 90 K, D_c_ = 1.366 g/cm^3^, R_1_ = 0.0265 (wR_2_ = 0.0692). Deposition number: CCDC 2088888. These data can be obtained free of charge from The Cambridge Crystallographic Data Centre via www.ccdc.cam.ac.uk/data_request/cif (accessed on 27 January 2022) or from the Cambridge Crystallographic Data Centre, 12 Union Road, Cambridge CB2 1EZ, UK; fax: +44-1223-336033; email: deposit@ccdc.cam.ac.uk. White crystals mp = 78 °C, **^1^H NMR (400 MHz, CDCl_3_)** δ 6.68 (s, 1H), 5.36 − 5.30 (m, 1H), 5.06 (t, *J* = 2.3 Hz, 1H), 4.77 − 4.72 (m, 2H), 3.32 − 3.22 (m, 1H), 3.17 (dd, *J* = 17.1, 3.7 Hz, 1H), 2.69 − 2.57 (m, 1H), 2.30 − 2.20 (m, 1H), 1.94 (td, *J* = 7.5, 3.8 Hz, 1H), 1.25 − 1.14 (m, 2H), 1.08 (s, 3H); **^13^C NMR (101 MHz, CDCl_3_)** δ 170.30, 150.00, 149.78, 149.48, 124.31, 123.09, 107.01, 62.05, 55.42, 40.16, 26.54, 22.56, 22.22, 21.65, 17.21, **MS (ESI+):** C_15_H_16_O_3_ [M + H]^+^: 245.17;

**Eucannabinolide (5)** white amorphous solid; **^1^H NMR (400 MHz, CDCl_3_)** δ 6.90 (t, *J* = 5.7 Hz, 1H), 6.35 (d, *J* = 2.2 Hz, 1H), 5.98 − 5.90 (m, 1H), 5.78 (d, *J* = 2.0 Hz, 1H), 5.29 − 5.23 (m, 2H), 5.23 − 5.16 (m, 2H), 4.39 (d, *J* = 5.8 Hz, 2H), 4.37 − 4.26 (m, 2H), 2.99 (s, 1H), 2.87 (s, 1H), 2.80 − 2.67 (m, 2H), 2.46 (d, *J* = 14.2 Hz, 1H), 2.34 − 2.24 (m, 1H), 2.12 (s, 3H), 1.86 − 1.74 (m, 6H); **^13^C NMR (101 MHz, CDCl_3_)** δ 170.27, 169.91, 165.74, 145.19, 137.42, 136.83, 135.59, 131.53, 126.28, 125.56, 125.23, 79.37, 76.97, 76.06, 59.28, 57.15, 48.68, 43.56, 29.60, 23.25, 21.33, 19.56; **MS (ESI+):** C_22_H_28_O_8_ [M+Na]^+^: 443.15.

### 3.6. Antileukemic Assays

MV4-11 AML cells were seeded onto 96-well plates and kept at a concentration of 0.5 million cells/mL. To determine the cell concentration, the cells were stained with Trypan Blue (0.4% Life Technologies, Carlsbad, CA, USA) and counted using the Cell Countess Invitrogen system. Cells were treated with *Magnolia grandiflora* extracts at concentrations of 0.1, 0.25, 0.5, 1, 2.5, 5, 10, 20, and 40 g/mL, in duplicate. After 48 h of treatment, cell viability was determined by flow cytometry. The cells were stained with YO-PRO-1 (Invitrogen-Thermo Fisher, Waltham, MA, USA) and 7-aminoactinomycin (7-AAD, Invitrogen-Thermo Fisher, Waltham, MA, USA) to assess viability. At least 10,000 events were recorded per condition on an LSR-Fortessa flow cytometer (BD Biosciences, Franklin Lakes, NJ, USA). Data analysis was conducted using the FlowJo 9.6 software for Mac OS X (TreeStar, Woodburn, OR, USA). Cells that were negative for YO-PRO-1 and 7-AAD were scored as viable. This same process was repeated with compounds **1**–**5**, except that viability was determined after treatment by 36 h. All compounds, except for compound **3**, were tested at concentrations of 1.25, 2.5, 5, 10, and 20 µM. Compound **3** was tested at concentrations of 1.25, 2.5, 5, 10, 12.5 20, 25, 50, 100, and 200 µM. 

### 3.7. Evaluation of Compounds in Normal Cells

Peripheral blood mononuclear cells (PBMCs) were isolated from the buffy coats of adult healthy volunteers (NPB 177, NPB 178, NPB 179). Cells were cultured in IMDM, 20% fetal bovine serum (FBS), and 10% penicillin–streptomycin (Life Technologies, Waltham, MA, USA) in 96-well plates. Cells were treated with compounds 1, 4, and 5 at concentrations of 2.5, 5, 10, and 20 µM, in triplicate. After 48 h of treatment, cell viability was determined using YO-PRO-1 and 7-AAD. At least 10,000 events were recorded per condition on an LSR-II flow cytometer (BD Biosciences, Franklin Lakes, NJ, USA). Data analysis was conducted using FlowJo 10.7.1 software for Mac OS X. Cells that were negative for YO-PRO-1 iodide and 7-AAD were scored as viable

## 4. Conclusions

We proposed an approach that facilitated the discovery of anticancer compounds from plants based on the hypothesis that those compounds were primarily present in a plant species to provide that species with an ecological advantage over other plant species. The approach was optimized using *Magnolia grandiflora*, a plant species known to have both antileukemic compounds and plant inhibitory activity. The method was used to correctly identify compound **1** as the major antileukemic compound in plant extracts, and it was also established that both the plant inhibitory activity and the antileukemic activity were correlated with the amount of compound **1** in the extracts. The method was applied to *Gynoxys verrucosa*, *Picradeniopsis oppositifolia*, and *Hedyosmum racemosum*, and in every case, the plant growth inhibitory activity of the extracts correlated with their antileukemic activity. The method was also able to rapidly locate the major antileukemic compounds (**1**, **2**, **4**, and **5**) in every extract. Overall, the growth inhibitory activities of the purified compounds showed a significant correlation with their antileukemic activities.

Compound **3**, which was inactive against leukemia cells (Figure 6) but was structurally very similar to the potent antileukemic compound **2**, had no plant growth inhibitory activity (Figure 7). The lack of plant growth inhibitory activity of compound **3** was likely due to the absence of an α,β-unsaturated exocyclic double bond in the lactone ring, as was the case in our previous study of its antileukemic activity [43].

Compounds **1** and **2**, as well as other sesquiterpene lactones, are known to inhibit the NF-κB stress response in human cells, which is critical for the survival of leukemia cells, and it has been suggested that this protein is involved in their mechanism of action [37,61,62]. In this mechanistic explanation, the sesquiterpene lactones interact with NF-κB via a nucleophilic attack by way of an α,β-unsaturated exocyclic double bond [63]. Although no plant NF-κB equivalent has been found [64], other proteins that are critical to the maintenance of both plant and leukemia cells, such as the MYB proteins, have been identified [65,66]. MYBs are conserved across all eukaryotic species, and a conserved MYB in leukemia called cMYB has been considered a promising target for therapy [67]. MYB proteins can be classified according to the presence of one or more repeating (“R”) MYB domains. Although the human 3R-MYB proteins regulate the G1/S transition, Feng et al. [68] discovered that the 3R-MYBs in plants functioned in both cell cycle regulation of the G1/S transition and the abiotic stress response, indicating that these proteins are critical to the adaptation and survival of sessile plants [68]. Compound **1** and related compounds have shown promising levels of activity against cMYB [69]. Given this information, it is plausible that 3R-MYB proteins in plants represent a target for growth inhibitory sesquiterpene lactones.

It is clear that the exocyclic α-methylene-γ-lactone is important for anticancer activity [29,43,62,70]. This was supported by the observation that compounds **1**, **2**, and **5** displayed activities against both plants and MV4-11 cells. Importantly, compound **3**, which lacks the exocyclic α-methylene-γ-lactone, was found to be inactive in both systems. This finding suggested that the proposed method had the ability to distinguish structurally related mechanistic features.

Although no mechanism of action has been proposed for compound **4**, the fact that it lacks the exocyclic α-methylene suggests that is likely to have anticancer and seed growth inhibitory effects via a different mechanism than compounds **1**, **2**, and **5**. This finding suggested that the current approach can be used to screen for multiple mechanistic pathways.

Importantly, the method was able to uncover growth inhibitory compounds that had activity against cancer cell lines but had low toxicity against normal cells.

Although plants have not developed bioactive molecules to cure human cancer, they have developed molecules that interfere with signaling pathways that are conserved across plants and humans. To our knowledge, the potential for the analysis of plant–plant interactions to discover antileukemic compounds or other types of anticancer compounds has not been demonstrated before. However, it has been suggested that the investigation of allelopathic interactions of marine organisms could be of value for the discovery of cytotoxic compounds [71]. A review of the literature, summarized in Table 1, suggests that the application of our approach could have helped to uncover many of the FDA-approved anticancer compounds of natural origin. Nevertheless, further experimentation is necessary to establish the generality and applicability of the method to discover compounds that are active against other types of cancer besides leukemia. The proposed approach can be implemented inexpensively in many parts of the world with rich biodiversity that may lack the infrastructure or resources necessary to implement the currently used high-throughput bioassay-directed isolation paradigms. The evidence presented in this paper shows that the investigation of plant–plant interactions may lead to the discovery of compounds with unique mechanisms of inhibition, and these discoveries may lead to future cancer therapies.

## Figures and Tables

**Table 1:** Inhibitory plant activities of clinically used, naturally occurring compounds or their precursors.

Anticancer Drug	Anticancer Activity	Plant Inhibition Activity
Paclitaxel	Paclitaxel stabilized microtubes in cancer cells and arrested the replication of cancer cells [16].	Paclitaxel arrested onion and maize root cells from dividing by stabilizing microtubules [72,73].
Vinblastine	Vinblastine destabilized microtubules in cancer cells and arrested replication [74].	Vinblastine bound to microtubules and created abnormal multipolar division in *Allium cepa* L. [73].
Podophyllotoxin, a precursor of etoposide and teniposide	Podophyllotoxin inhibited microtubule organization in cancer cells [75]. Etoposide killed cancer cells by inhibiting topoisomerase II (TopoII) [76].	Podophyllotoxin inhibited onion (*Allium cepa* L.) root growth by affecting the formation of mitotic microtubular organizing centers [77]. Etoposide inhibited the division of polyploid cells of grass pea (*Lathyrus sativus* L.) seedlings. The presumed binding regions of etoposide to TopoII were conserved in plants, *Drosophila melanogaster*, yeast, and humans [78].
Camptothecin, a precursor to irinotecan and topotecan	Camptothecin killed cancer cells by inhibiting topoisomerase 1 [16].	Camptothecin selectively caused the inhibition of young developing vascular tissues of the axillary buds of *Nicotiana tabacum* L. Camptothecin inhibited the sprouting of potatoes by interfering with cell division in the meristem [79]. Early reports showed this using a partially purified enzyme from barley seeds, and strong inhibition of the relaxation of supercoiled pBR322 DNA by the barley DNA enzyme was observed with camptothecin [79]. Later work showed that plants contained a conserved Topo1, and camptothecin-producing plants, including *Camptotheca acuminata*, *Ophiorrhiza pumila*, and *Ophiorrhiza liukiuensis*, had point mutations in Topo1 that conferred resistance to autotoxicity [80].
Homoharringtonine	Homoharringtonine was used for tyrosine kinase inhibitor-resistant chronic myelogenous leukemia (CML). It worked by binding to the A-site of the 80S ribosome and inhibiting translation [81].	Harringtonine alkaloids, which are related to homoharringtonine, had plant growth regulating activity [79]. The 80S ribosome was conserved across species [82].
Maytansine, a precursor to trastuzumab-emtansine.	Maytansine bound to β-tubulin and blocked the formation of longitudinal tubulin interactions in microtubules [83].	Maytansine inhibited growth in tobacco callus (*Nicotiana tabacum* L.) and rice seedling bioassays [79].
Ellipticine, a precursor to elliptinium	Elliptinium is approved in France for the treatment of metastatic breast cancer. Elliptinium and ellipticine inhibited topoisomerase II [84].	Ellipticine potently inhibited mungbean hypocotyls (Chen, Witham). Ellipticine has been postulated to bind to the same regions of TopoII as etoposide [85]. These regions were conserved in plants, *Drosophila melanogaster*, yeast, and humans [78].

## Supplementary Materials

The following supporting information can be downloaded at: https://www.mdpi.com/article/10.3390/molecules27092928/s1, Figure S1: Growth inhibition of extracts on *Lolium perenne* and *Lactuca sativa*; Figure S2: TLC Bioautography of MGME; Figure S3: TLC Bioautography of POME; Figure S4: Root inhibition and antileukemic activity correlation of sesquiterpene lactones; Figure S5: ^1^H NMR of Parthenolide (1); Figure S6: ^13^C NMR of Parthenolide (1); Figure S7: ^1^H NMR of Dehydroleucodine (2); Figure S8: ^13^C NMR of Dehydroleucodine (2); Figure S9: ^1^H NMR of Leucodine (3); Figure S10: ^13^C NMR of Leucodine (3); Figure S11: ^1^H NMR of Onoseriolide (4); Figure S12: ^13^C NMR of Onoseriolide (4); Figure S13: ^1^H NMR of Eucannabinolide (5); Figure S14: ^13^C NMR of Eucannabinolide(5).

## Sample Availability

Samples of the compounds **1**–**5** are available from the authors.
