# Supplementary material for: Analysis of Plant–Plant Interactions Reveals the Presence of Potent Antileukemic Compounds
Source: Molecules. 2022 May 4;27(9):2928. doi: 10.3390/molecules27092928 (PMC9105371; doi:10.3390/molecules27092928)
Supplement: Supplementary file 1 [file molecules-27-02928-s001.zip › molecules-1595345-supplementary.pdf]

Supporting Information

Analysis of Plant–Plant Interactions Reveals the Presence of Potent Antileukemic Compounds

David E. Mery [1,2], Amanda J. Compadre [1], Paola E. Ordóñez [3], Edward J. Selvik [1], Vladimir Morocho [4], Jorge Contreras [5], Omar Malagón [4], Darin E. Jones [1], Philip J. Breen [1], Michael J. Balick [6], Flavio G. Gaudio [7], Monica L. Guzman [5,*] and Cesar M. Compadre [1,*]

[1] Department of Pharmaceutical Sciences, University of Arkansas for Medical Sciences, Little Rock, AR 72205, USA; dmery@uams.edu (D.E.M.); amanda.compadre@ucsf.edu (A.J.C.); eselvik@uams.edu (E.J.S.); dejones@uams.edu (D.E.J.); breenphilipj@uams.edu (P.J.B.);
[2] SeqRX, LLC, Little Rock, AR 72205, USA
[3] School of Chemical Sciences and Engineering, Yachay Tech University, Urcuquí 100119, Ecuador; pordonez@yachaytech.edu.ec
[4] Departamento de Química, Universidad Técnica Particular de Loja, San Cayetano Alto s/n, Loja 1101608, Ecuador; svmorocho@utpl.edu.ec (V.M.); omalagon@utpl.edu.ec (O.M.)
[5] Department of Medicine, Division of Hematology/Oncology, Weill Cornell Medical College, New York, NY 10021, USA; joc4006@med.cornell
[6] Institute for Economic Botany, New York Botanical Garden, New York, NY 10458, USA; mbalick@nybg.org
[7] Department of Emergency Medicine, New York Presbyterian-Weill Cornell Medicine, New York, NY 10065, USA; flg9002@med.cornell.edu

* Correspondence: mlg2007@med.cornell.edu (M.L.G.); compadrecesarm@uams.edu (C.M.C.); Tel.: +1-212-746-6838 (M.L.G.); +1-501-993-4972 (C.M.C.)

Abstract: A method to identify anticancer compounds in plants was proposed based on the hypothesis that these compounds are primarily present in plants to provide them with an ecological advantage over neighboring plants and other competitors. According to this view, identifying plants that contain compounds that inhibit or interfere with the development of other plant species may facilitate the discovery of novel anticancer agents. The method was developed and tested using Magnolia grandiflora, Gynoxys verrucosa, Picradeniopsis oppositifolia, and Hedyosmum racemosum, which are plant species known to possess compounds with cytotoxic activities. Plant extracts were screened for growth inhibitory activity, and then a thin-layer chromatography bioautography assay was conducted. This located the major antileukemic compounds 1, 2, 4, and 5 in the extracts. Once the active compounds were located, they were extracted and purified, and their structures were determined. The growth inhibitory activity of the purified compounds showed a significant correlation with their antileukemic activity. The proposed approach is rapid, inexpensive, and can easily be implemented in areas of the world with high biodiversity but with less access to advanced facilities and biological assays.

Table of Contents

Growth inhibition of extracts on Lolium perenne and Lactuca sativa.	Figure S1
TLC Bioautography of MGME	Figure S2
TLC Bioautography of POME	Figure S3
Root inhibition and antileukemic activity correlation of sesquiterpene lactones	Figure S4
^{1}H NMR of Parthenolide (1)	Figure S5
^{13}C NMR of Parthenolide (1)	Figure S6
^{1}H NMR of Dehydroleucodine (2)	Figure S7
^{13}C NMR of Dehydroleucodine (2)	Figure S8
^{1}H NMR of Leucodine (3)	Figure S9
^{13}C NMR of Leucodine (3)	Figure S10
^{1}H NMR of Onoseriolide (4)	Figure S11
^{13}C NMR of Onoseriolide (4)	Figure S12
^{1}H NMR of Eucannabinolide (5)	Figure S13
^{13}C NMR of Eucannabinolide (5)	Figure S14

General

Compound characterization

Spectra were recorded in CDCl3 at 25°C, on a Varian Unity NMR spectrometer, operating at 400 MHz for 1H and 100 MHz for 13C spectra, equipped with a One NMR W012 probe. Each sample consisted of approximately 5 to 10 mg of compound dissolved in 0.7 mL of CDCl3. All 1D and 2D (gradient COSY, NOESY, TOCSY, gradient HSQC and gradient HMBC) spectra were acquired and processed with MestReNova version 14.1.1. X-Ray Crystallography was performed on a Bruker-Nonius X8 Proteum CCD diffractometer using CuK (alpha) radiation. The structures were solved using SHELXT and refined using SHELXL. (Sheldrick, 2007) Molecular fragment editing was performed using the XP program of SHELXTL. Crystallographic data for all the structures was deposited in the Cambridge Crystallographic Data Centre. The data can be obtained free of charge via www.ccdc.cam.ac.uk or by contacting the Cambridge Crystallographic Data Centre, 12 Union Road, Cambridge CB2 1EZ, UK. fax

Figure S1: Growth Inhibition of whole extract of Lolium perenne and Lactuca sativa

Lolium perenne subsp. multiflorum (mg/mL) Lactuca sativa (mg/mL)

Extract	Germ	Seedling	Root	Shoot	Germ	Seedling	Root	Shoot
GVEA	0.69 ±0.095	0.25 ± 0.006	0.16 ± 0.034	0.40 ± 0.078	3.20 ± 0.55	1.33 ± 0.07	1.20 ± 0.06	1.34 ± 0.01
HRME	1.96 ± 0.19	0.61 ± 0.096	0.50 ± 0.155	1.07 ± 0.055	12.18 ± 0.38	3.60 ± 0.16	3.72 ± 0.13	3.33 ± 0.22
MGEA	1.52 ± 0.33	1.06 ± 0.12	0.82 ± 0.138	1.23 ± 0.062	2.65 ± 0.24	1.10 ± 0.05	1.05 ± 0.03	1.35 ± 0.06
MGME	>15	>15	11.8 ± 2.94	>15	>15	>15	>15	>15
POEA	>3	1.86 ± 0.28	0.88 ± 0.249	>3	>3	2.97 ± 0.66	1.91 ± 0.14	>3
POME	4.34 ± 0.44	1.99 ± 0.02	1.68 ± 0.071	2.73 ± 0.095	>15	8.75 ± 0.28	5.69 ± 0.78	12.4 ± 0.26

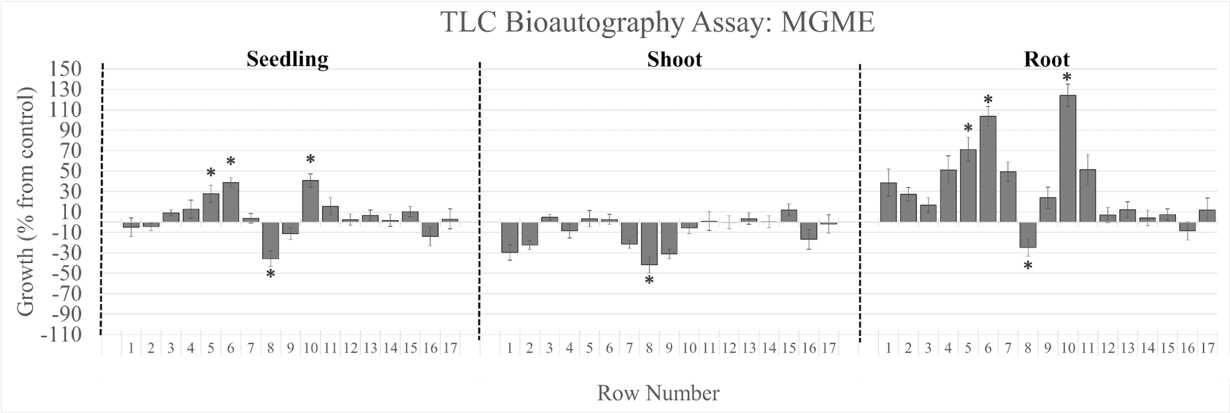

Figure S2: Bioautography of MGME

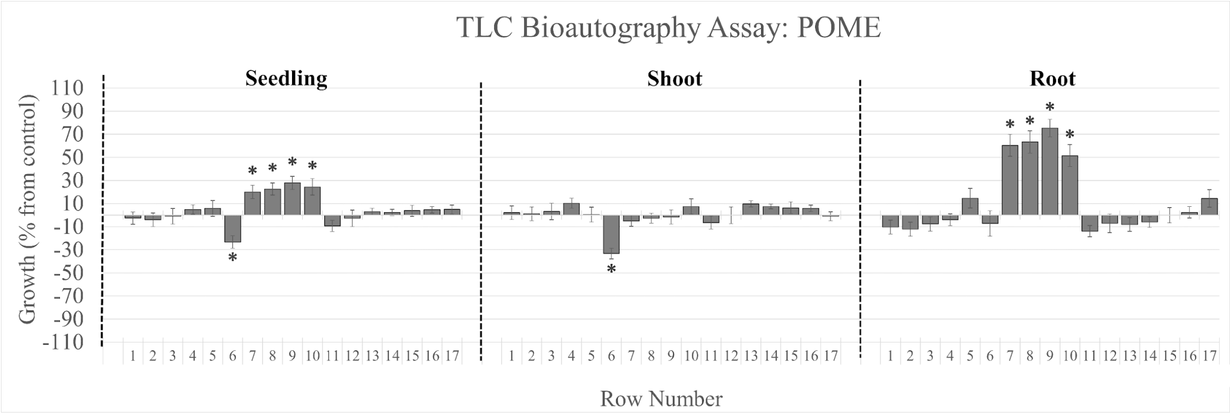

Figure S3: Bioautography of POME

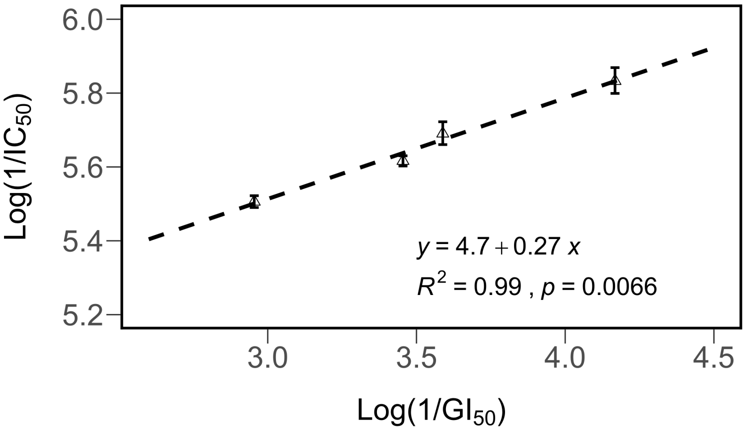

Figure S4: Root inhibition and antileukemic activity correlation

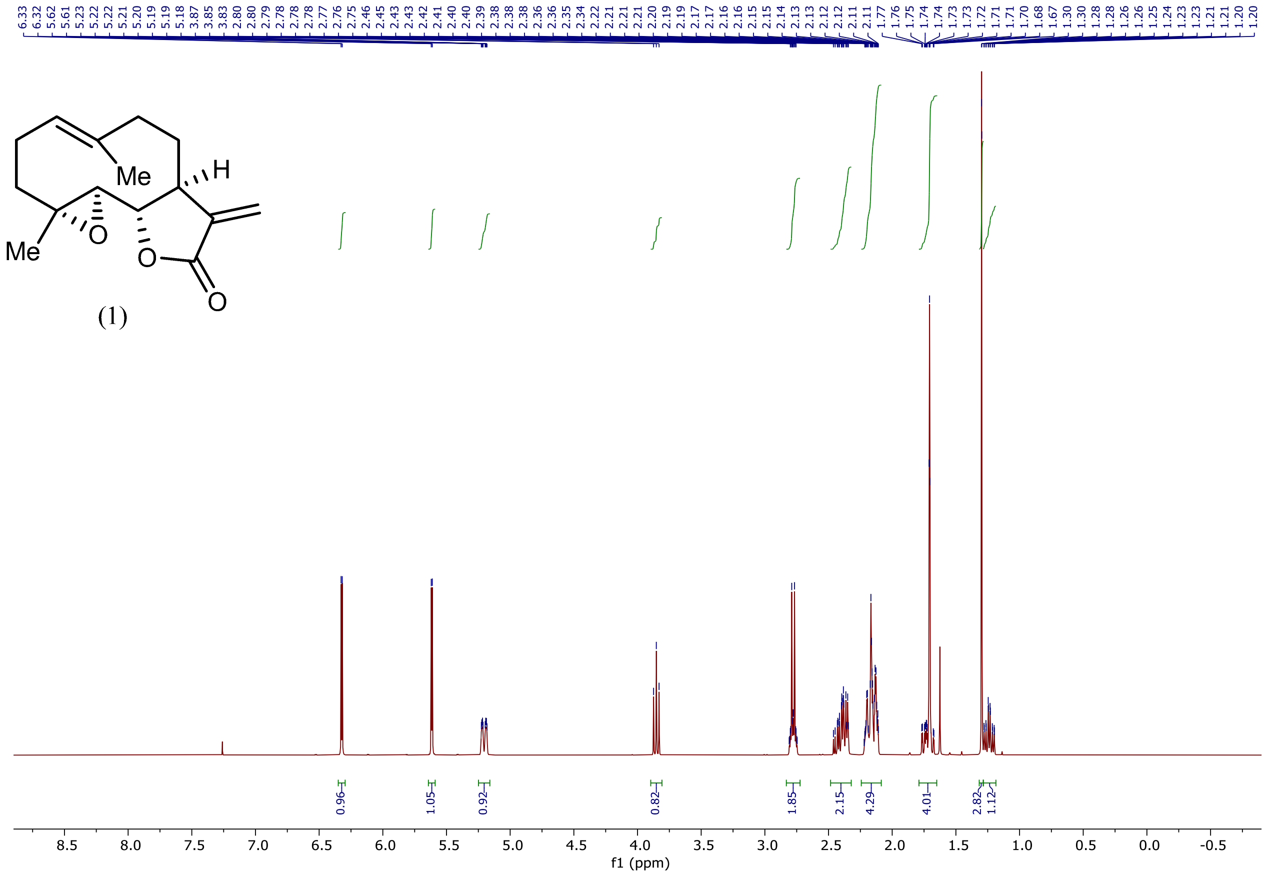

Figure S5: ^{1}H NMR of Parthenolide (1)

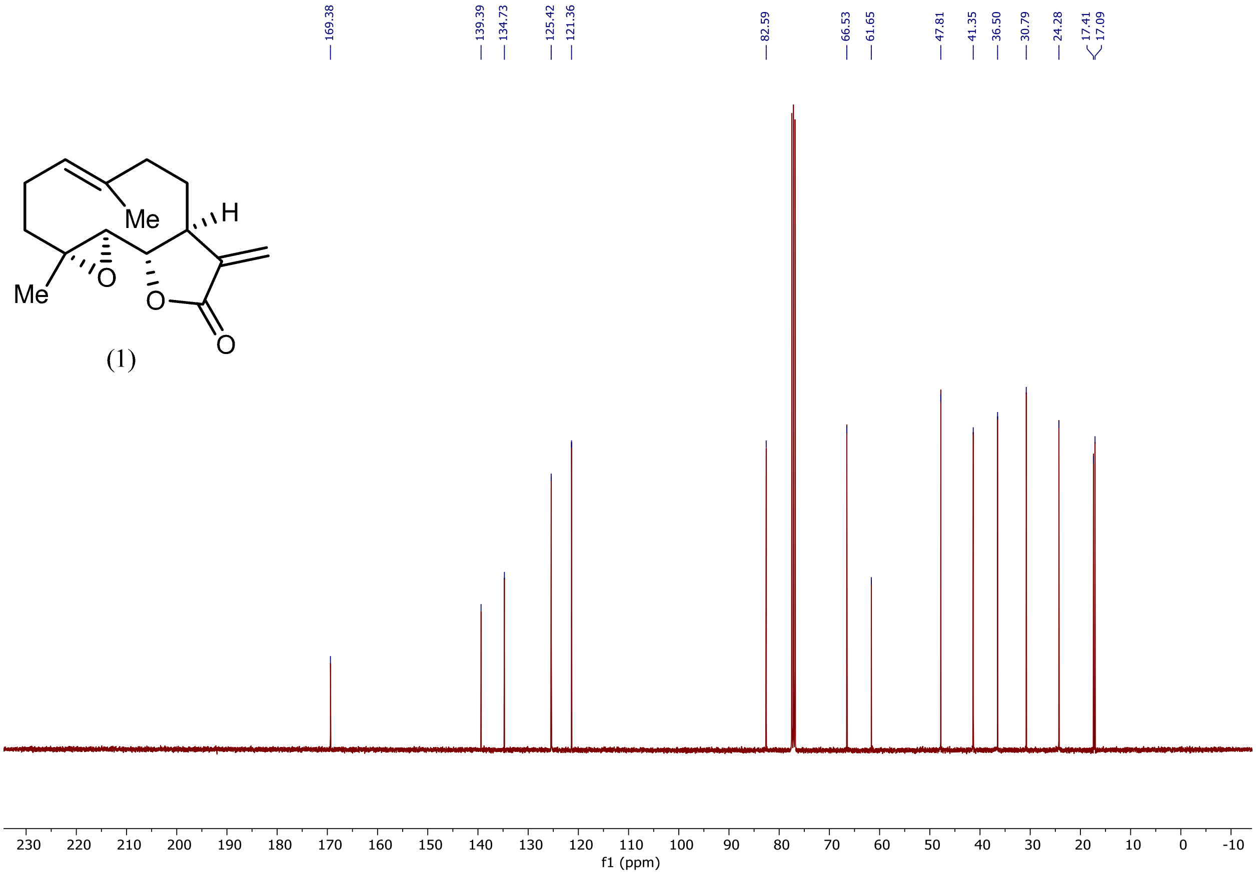

Figure S6: ^{13}C NMR of Parthenolide (1)

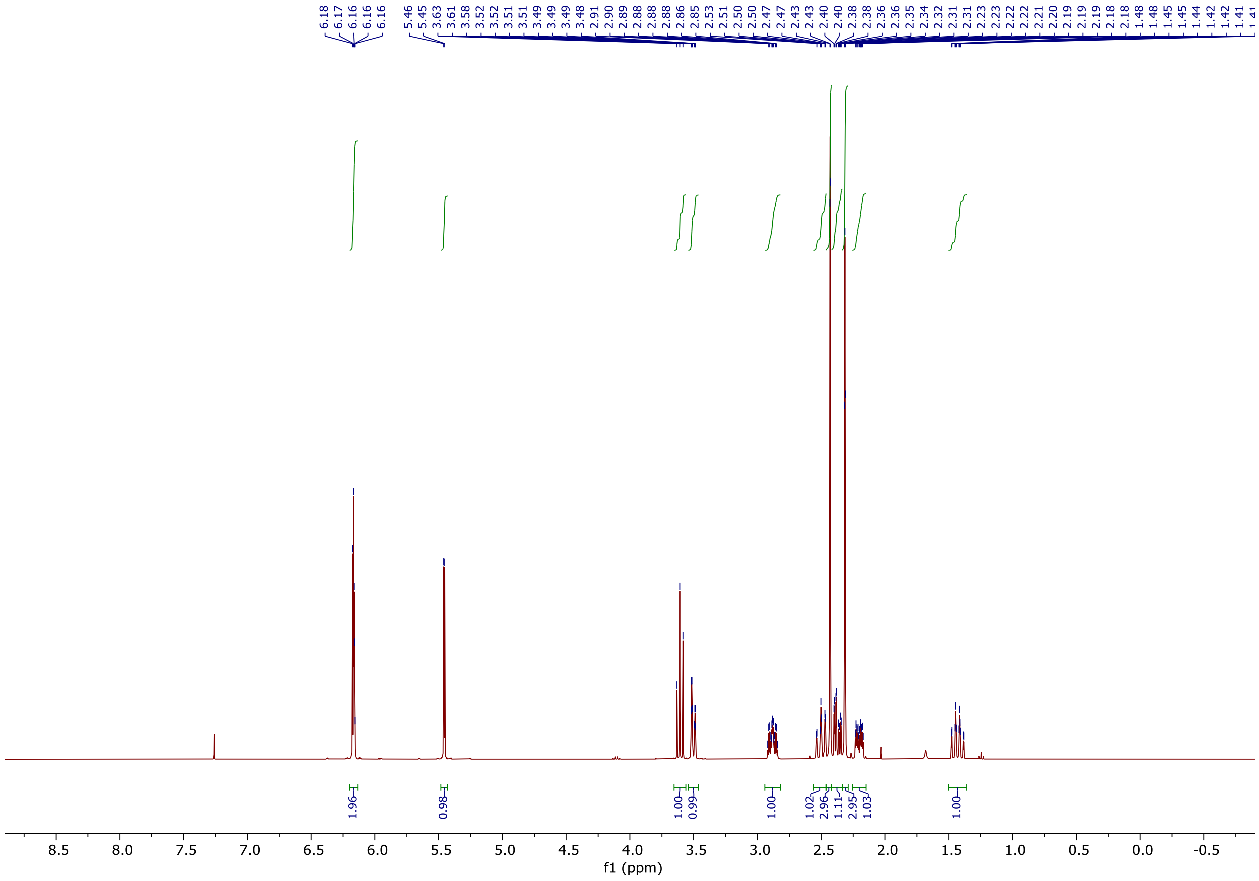

Figure S7: ^{1}H NMR of Dehydroleucodine (2)

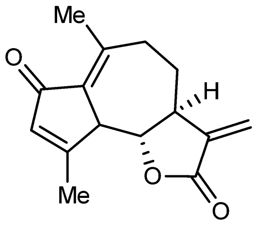

(2)

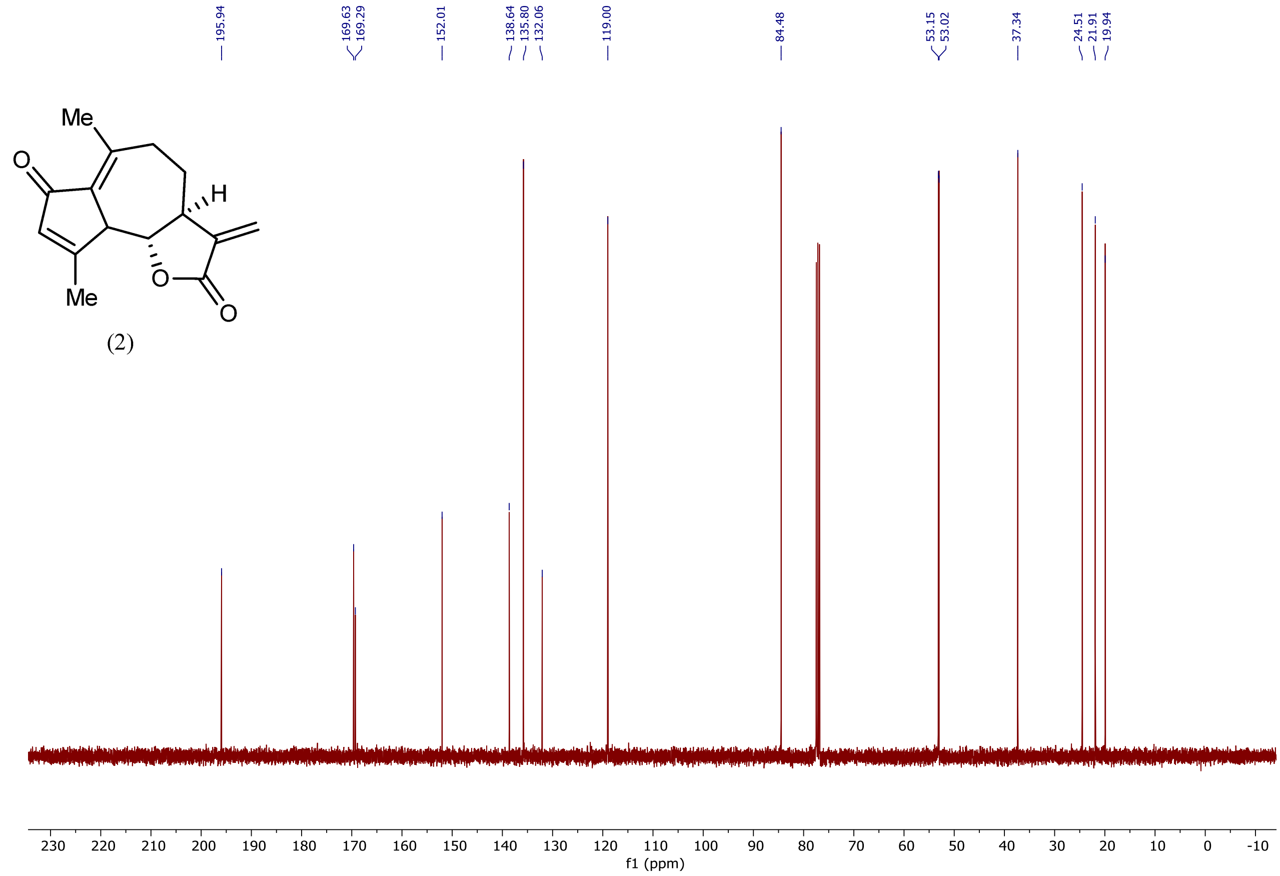

Figure S8: ^{13}C NMR of Dehydroleucodine (2)

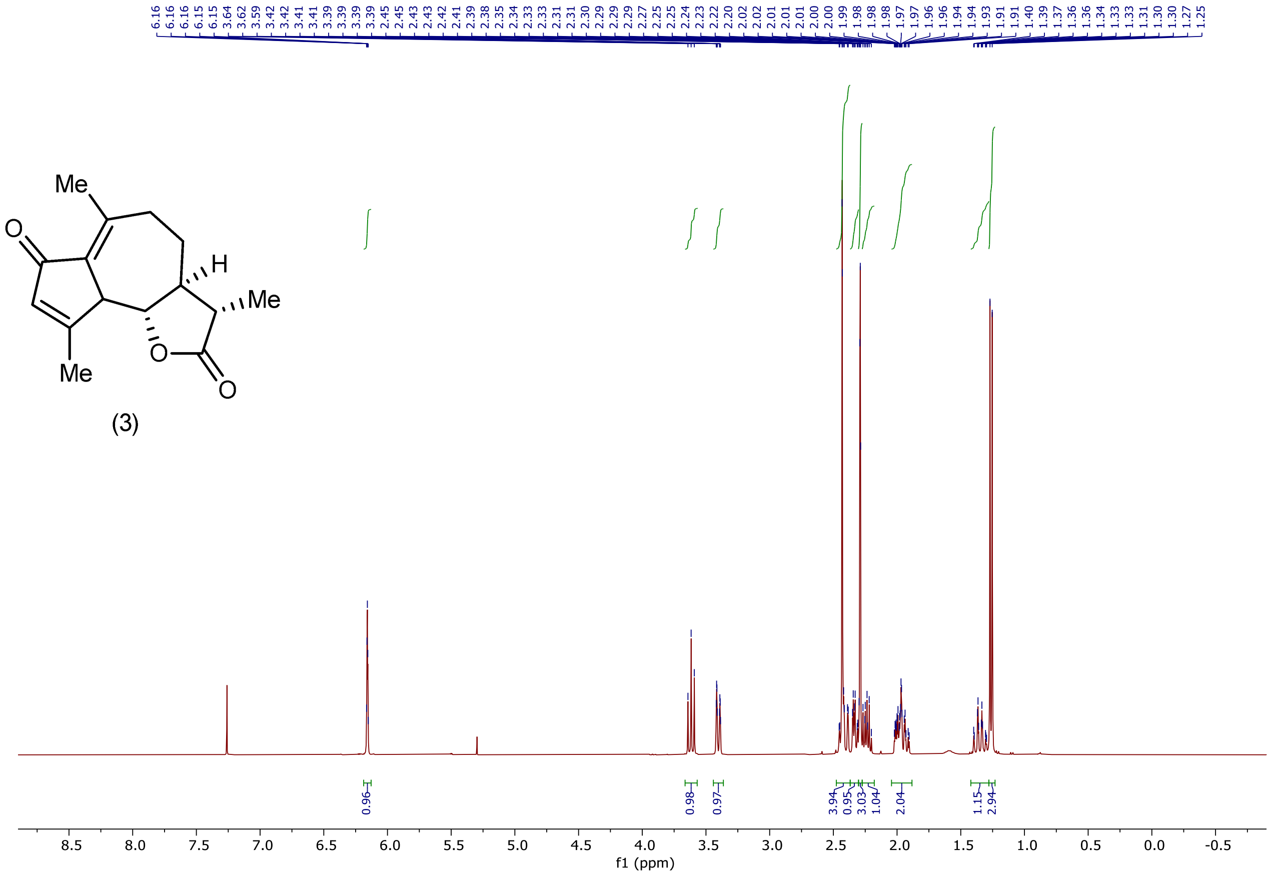

(3)

Figure S9: ¹H NMR of Leucodine (3

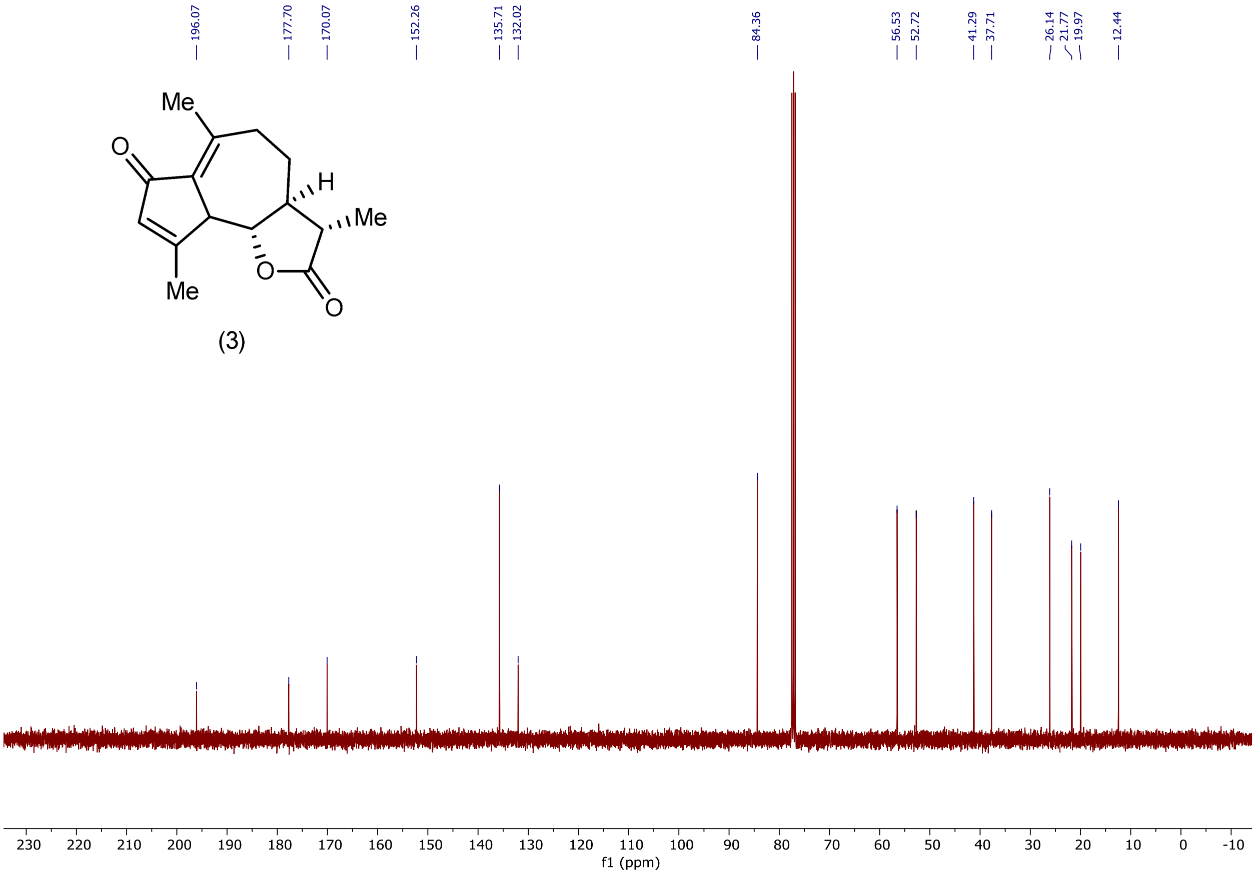

Figure S10: ^{13}C NMR of Leucodine (3)

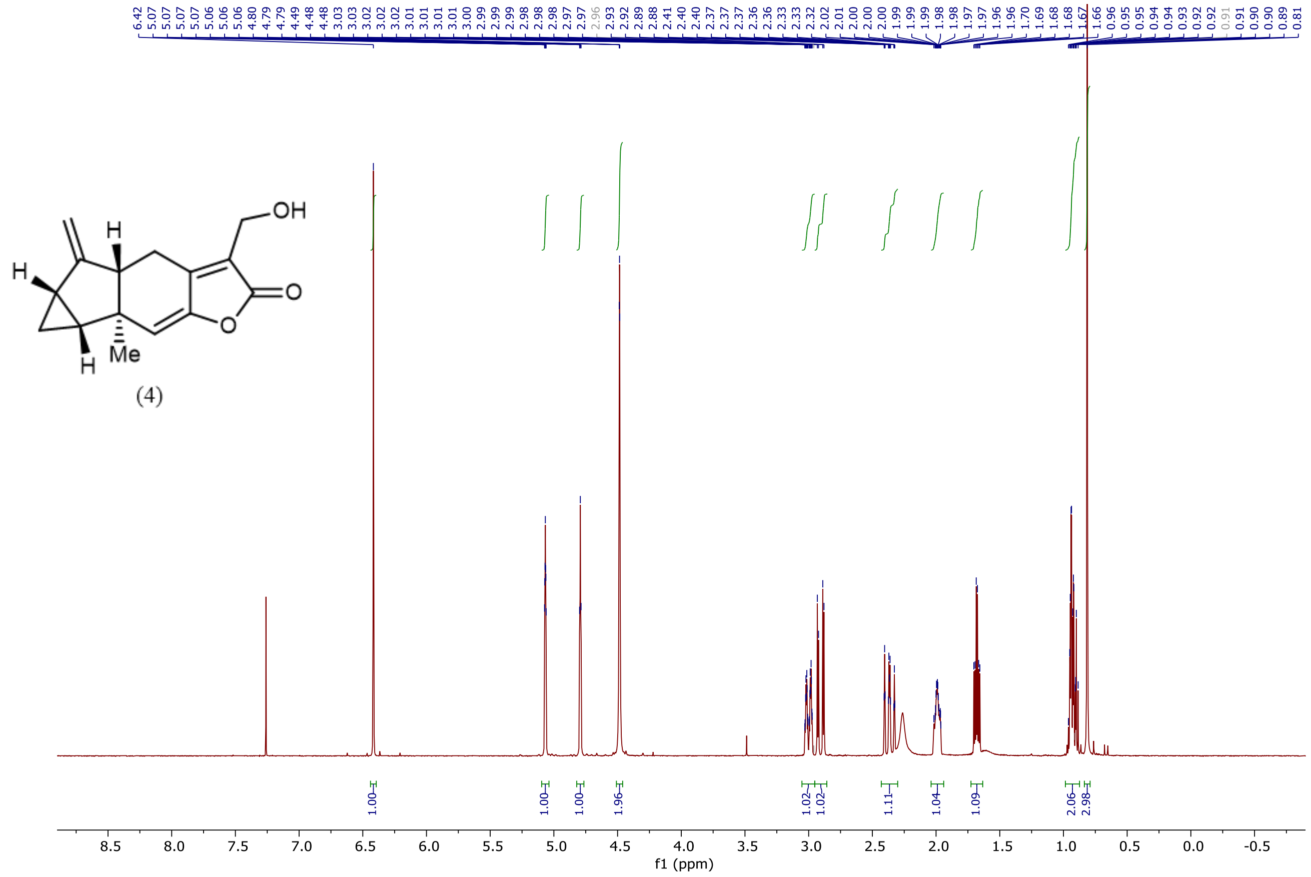

Figure S11: ^{1}H NMR of Onoseriolide (4)

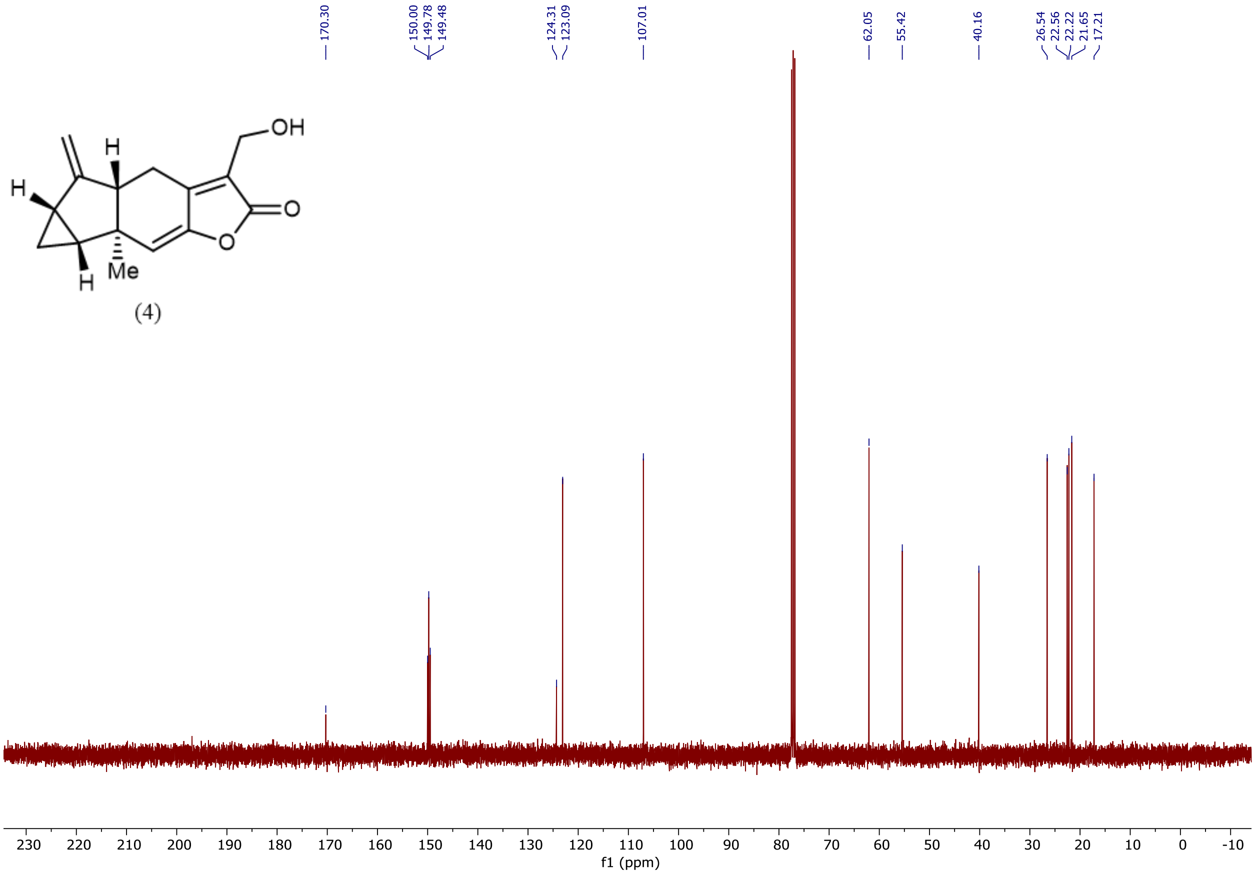

Figure S12: ^{13}C NMR of Onoseriolide

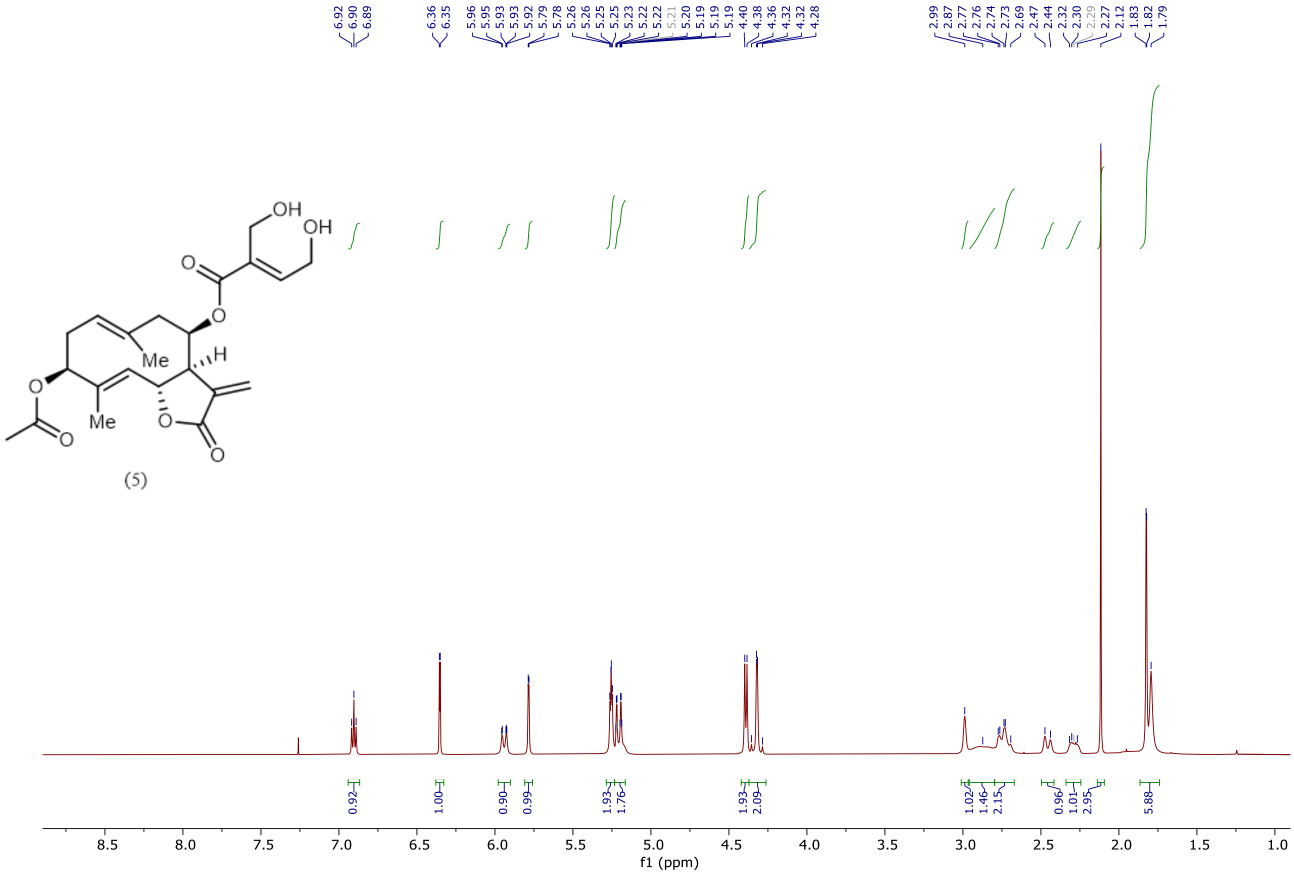

Figure S13: ¹H NMR of Eucannabinolide (5)

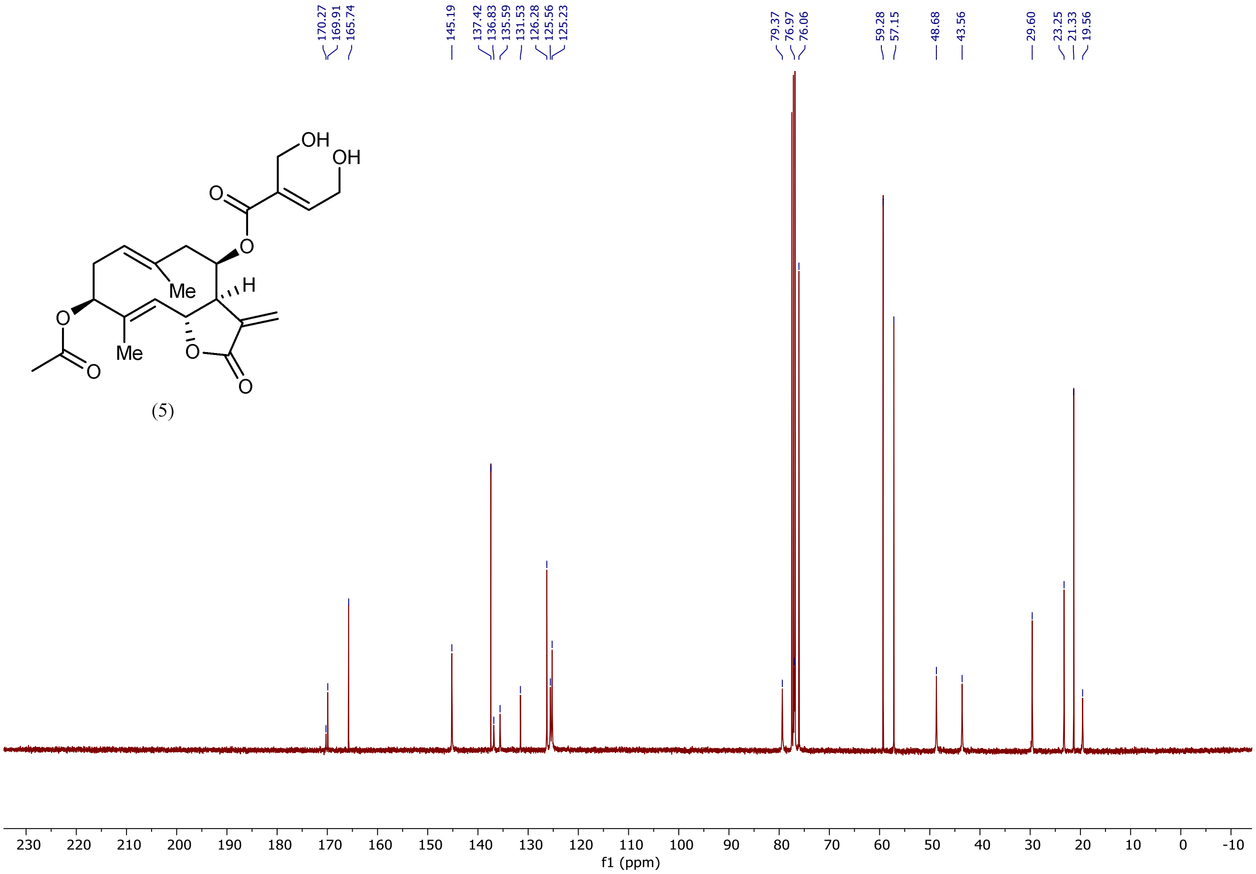

Figure S14: ^{13}C NMR of Eucannabinolide (5)
